# Supplementary material for: Polymorphic cobalt diselenide as extremely stable electrocatalyst in acidic media via a phase-mixing strategy
Source: Nat Commun. 2019 Nov 25;10:5338. doi: 10.1038/s41467-019-12992-y (PMC6877578; doi:10.1038/s41467-019-12992-y)
Supplement: Supplementary file 1 — Supplementary Information [file 41467_2019_12992_MOESM1_ESM.pdf]

Supplementary Information

Polymorphic cobalt diselenide as extremely stable electrocatalyst in acidic media via a phase-mixing strategy

Zhang et al.

Supplementary Figures

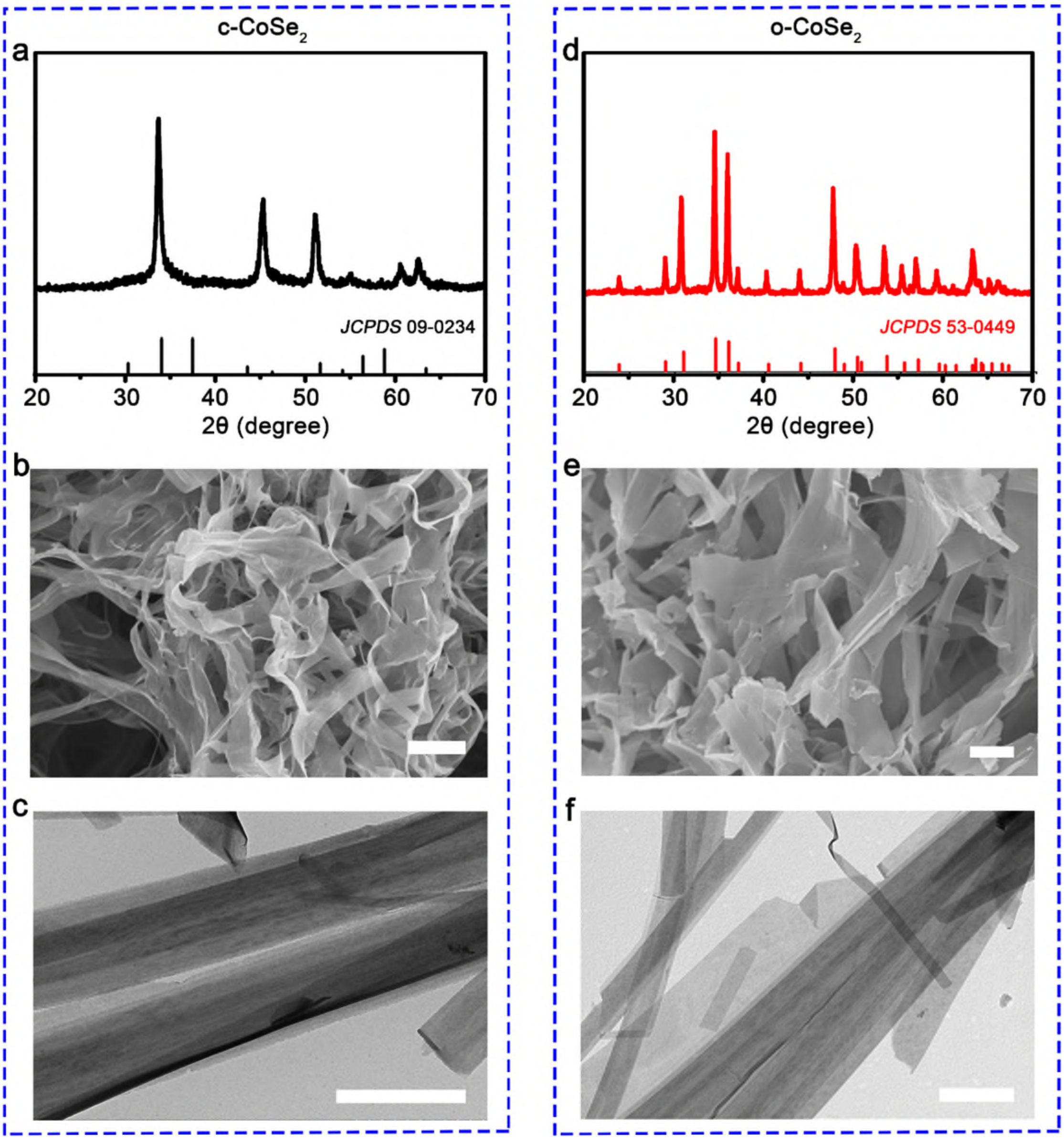

Supplementary Figure 1. Physical characterization of c-$CoSe_2$ and o-$CoSe_2$ nanobelts. a-c, XRD pattern (**a**), SEM (**b**) and TEM (**c**) of c-$CoSe_2$. **d-f,** XRD pattern (**d**), SEM (**e**) and TEM (**f**) of o-$CoSe_2$. XRD patterns in **a** and **d** show that the diffraction peaks can be perfectly assigned to the pyrite $CoSe_2$ with cubic phase (*JCPDS* 09–0234) and marcasite $CoSe_2$ with orthorhombic phase (*JCPDS* 53–0449), respectively. Scale bars: **b, e,** 1 μm and **c, f,** 500 nm.

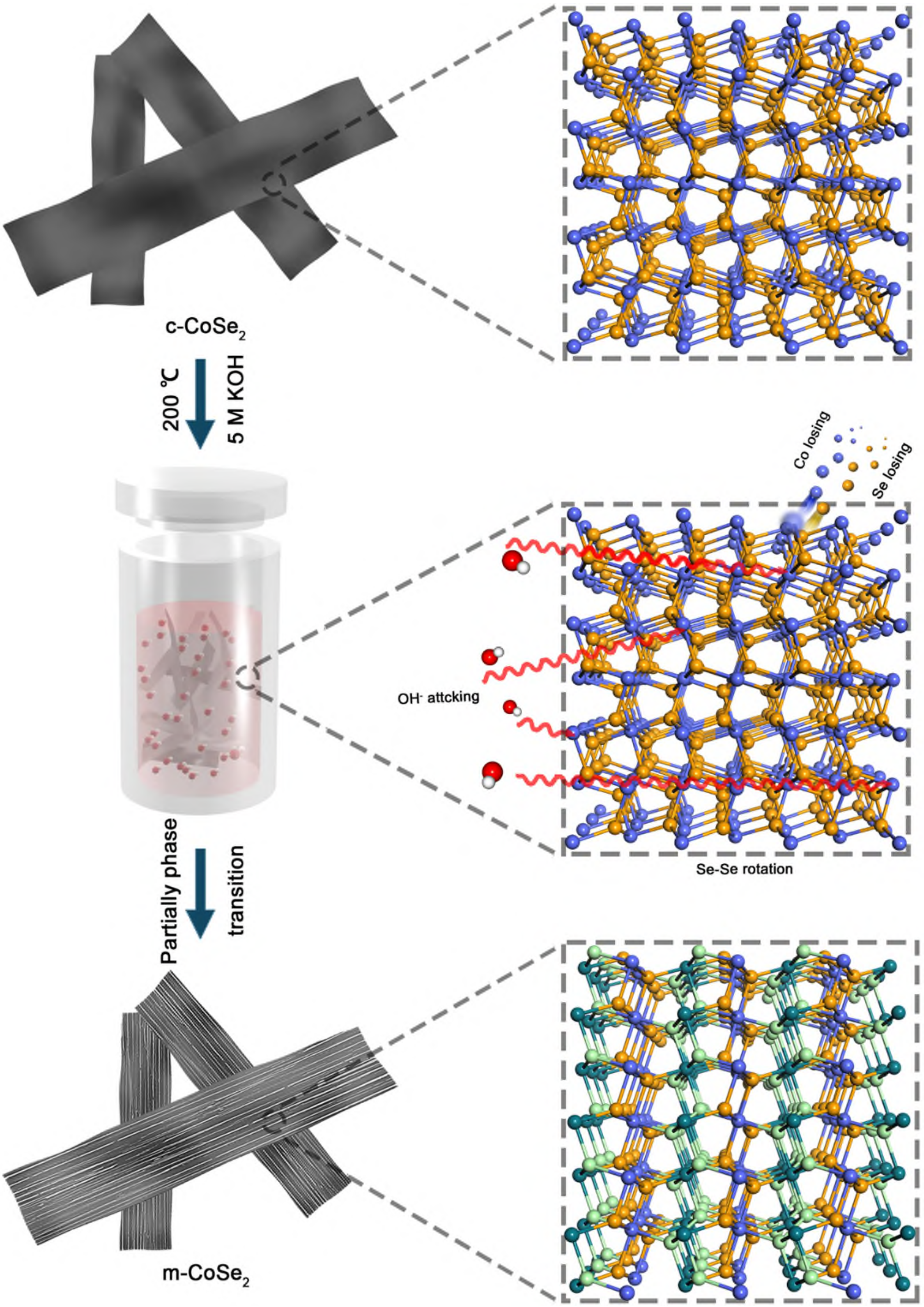

Supplementary Figure 2. Schematic illustration of the synthesis of m-$CoSe_2$ via alkali-heating method. This harsh condition causes the leaching of Co and Se at defective sites of c-$CoSe_2$, leaving atomic vacancies that mediate the structural phase transition of c-$CoSe_2$ to o-$CoSe_2$.

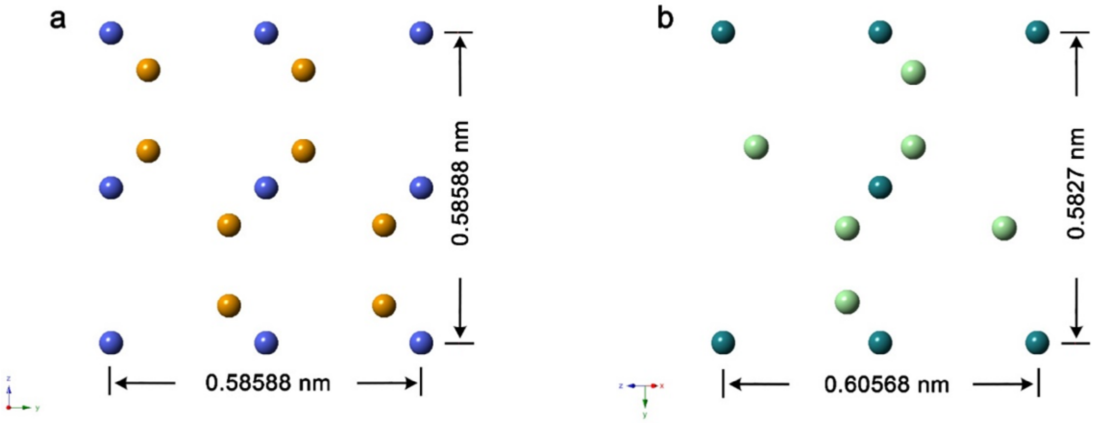

Supplementary Figure 3. Crystal model of the c-$CoSe_2$ and o-$CoSe_2$. a, b Atomic models of the c-$CoSe_2$(100) and o-$CoSe_2$ (101), respectively, which show that c-$CoSe_2$(100) and o-$CoSe_2$ (101) facets almost possess the same atomic arrangement.

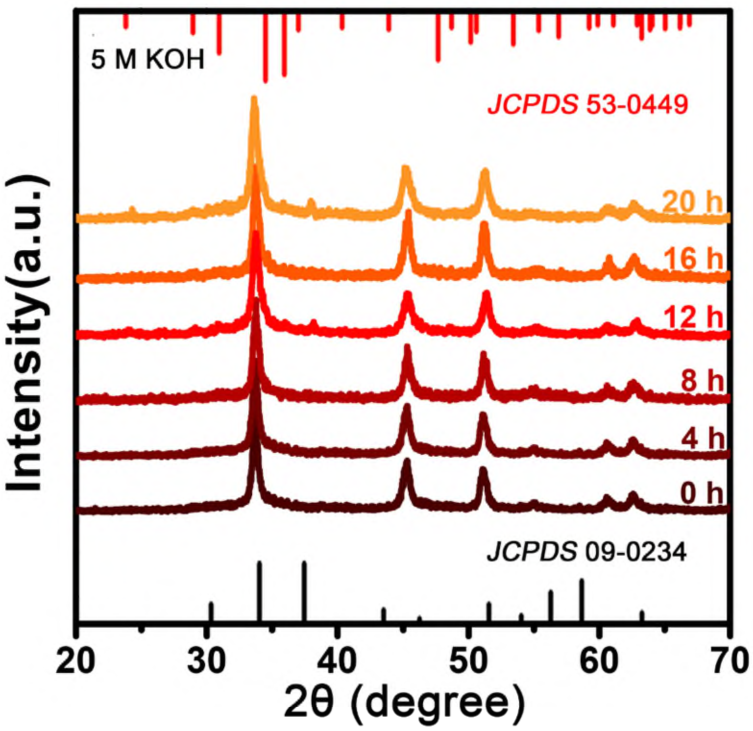

Supplementary Figure 4. XRD patterns. XRD patterns of the products achieved at room temperature for different reaction time, which exhibit that, at room temperature, the products are still cubic phase even the reaction time is up to 20 hours.

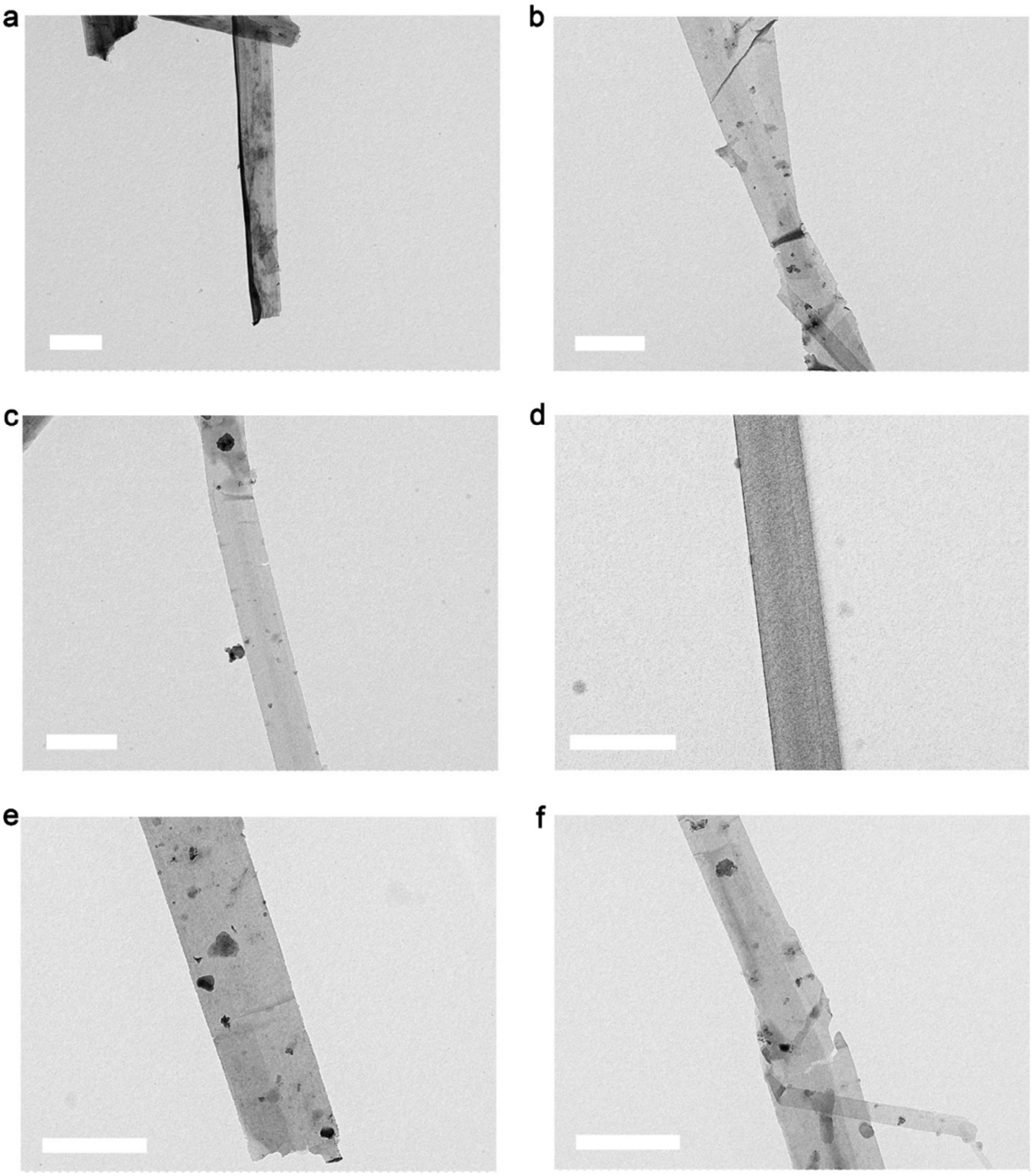

Supplementary Figure 5. TEM images. TEM images of the products achieved at room temperature for different reaction time: **a,** 0 h, **b,** 4 h, **c,** 8 h, **d,** 12 h, **e,** 16 h and **f,** 20 h, respectively. Scale bars, 500 nm.

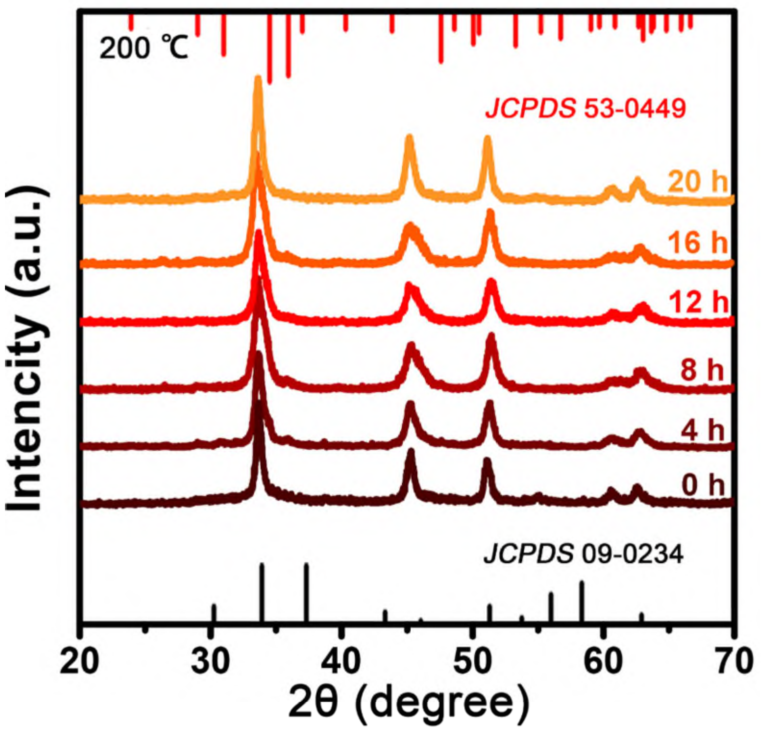

Supplementary Figure 6. XRD patterns. XRD patterns of the products synthesized at 200 °C for different reaction time without KOH, which exhibit that the obtained product is still cubic phase if no KOH was added in the synthesis.

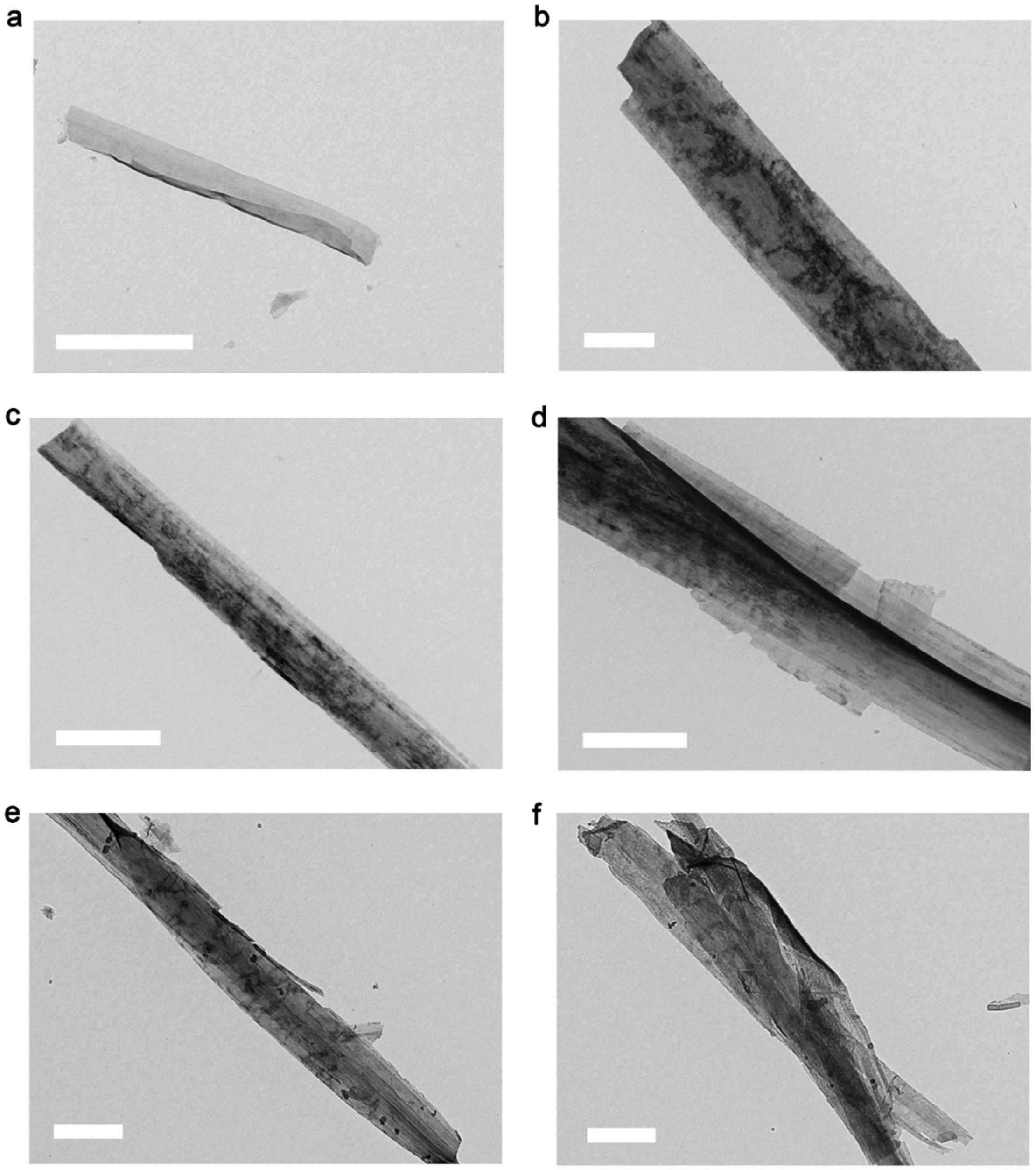

Supplementary Figure 7. TEM images. TEM images of the products obtained at 200 °C for different reaction time without KOH. **a,** 0 h, **b,** 4 h, **c,** 8 h, **d,** 12 h, **e,** 16 h and **f,** 20 h, respectively. Scale bars, 500 nm.

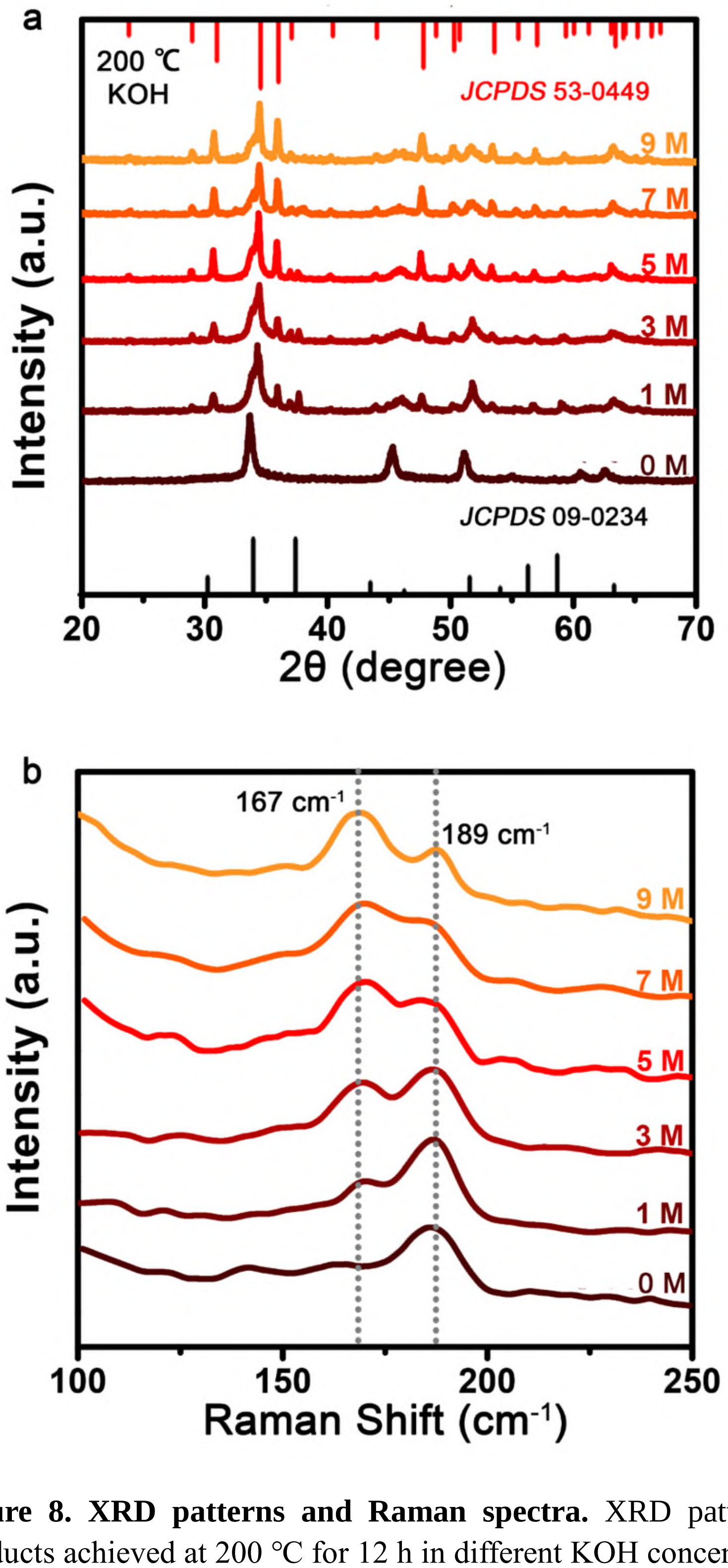

Supplementary Figure 8. XRD patterns and Raman spectra. XRD patterns (**a**) and Raman spectra (**b**) of the products achieved at 200 °C for 12 h in different KOH concentration solutions. The results reveal that the phase transition emerges with KOH in the synthesis when the temperature reaches 200 ºC.

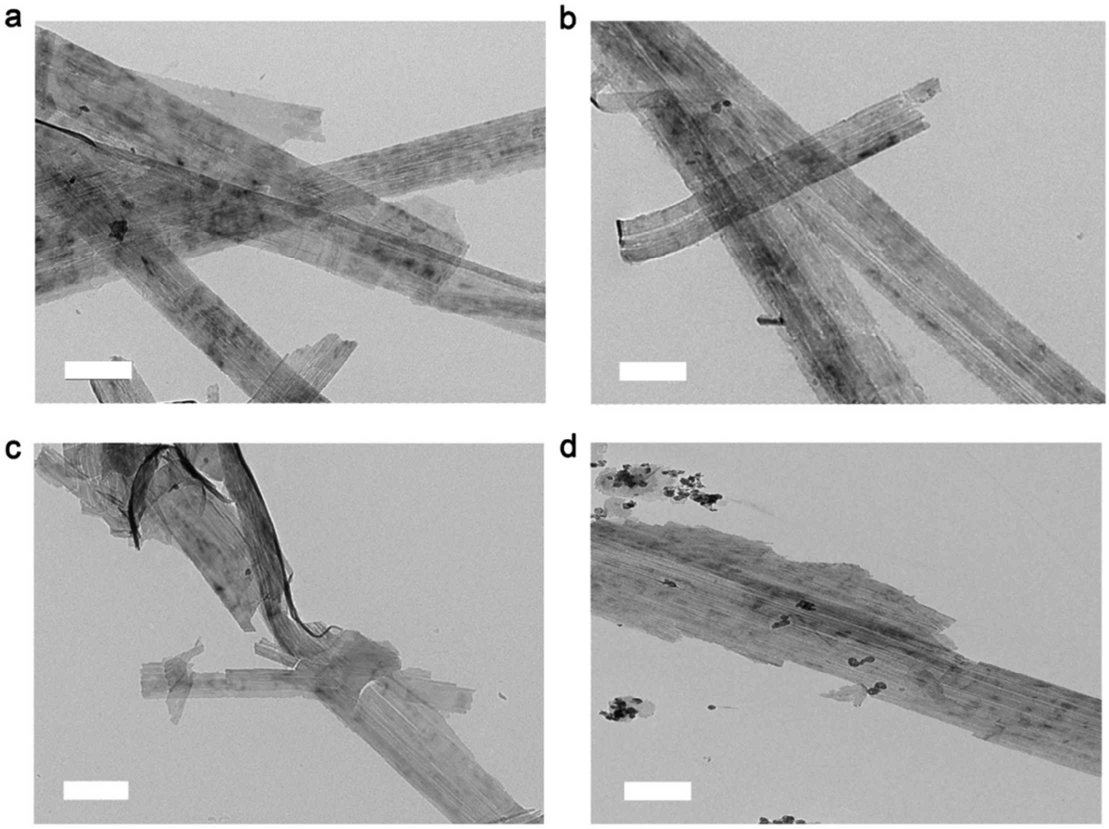

Supplementary Figure 9. TEM images. TEM images of the products achieved at 200 °C for 12 h in different KOH concentration solutions: **a,** 1 M, **b,** 3 M, **c,** 7 M and **d,** 9 M, respectively, Scale bars, 200 nm. Although in the harsh reaction condition, the m-$CoSe_2$ can keep decent belt-like morphology that inherited from c-$CoSe_2$ precursors, only some surface scratches on m-$CoSe_2$ were observed.

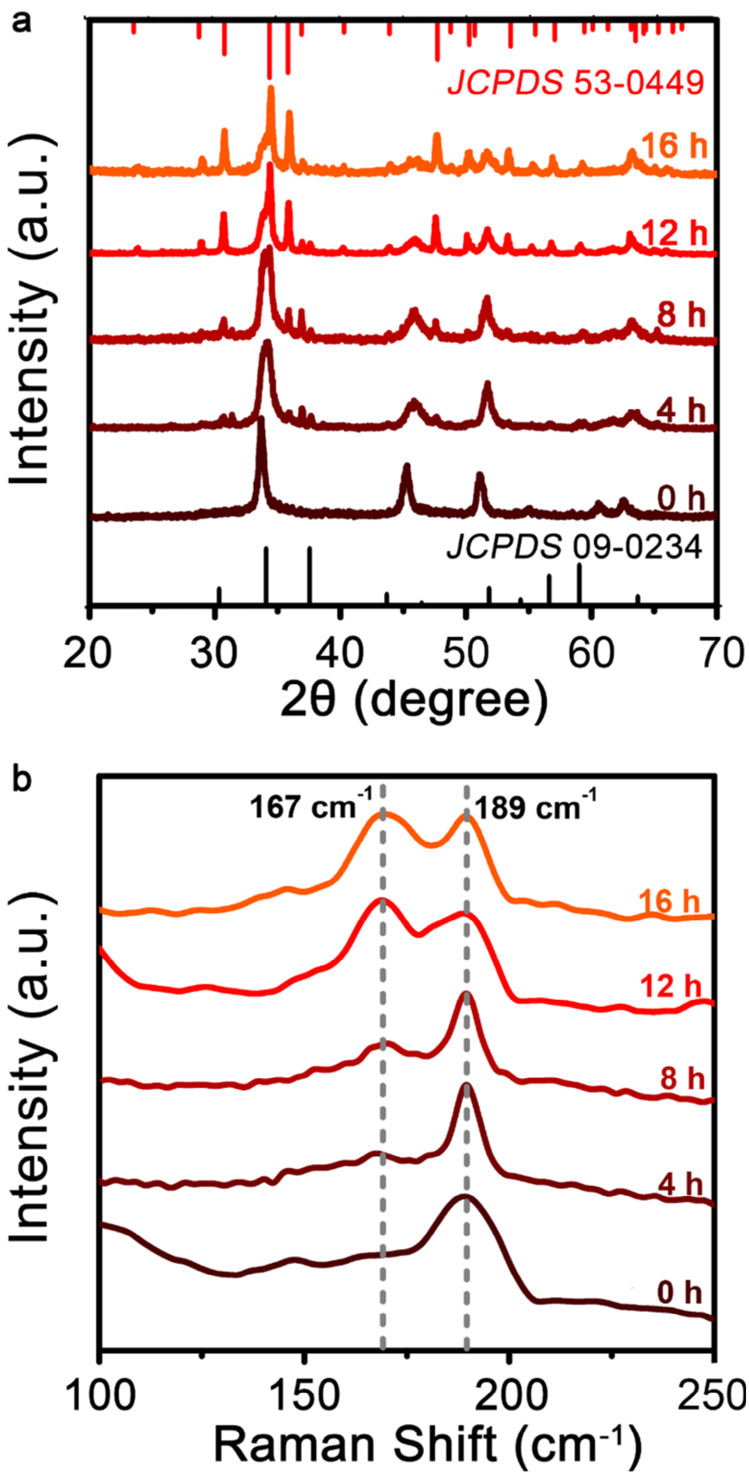

Supplementary Figure 10. XRD patterns and Raman spectra. XRD patterns (**a**) and Raman spectra (**b**) of the products achieved at 200 ^{o}C for different reaction time in 5 M KOH. The results reveal that the phase transition emerges at ~4 h when the temperature reaches 200 ^{o}C in 5 M KOH solution.

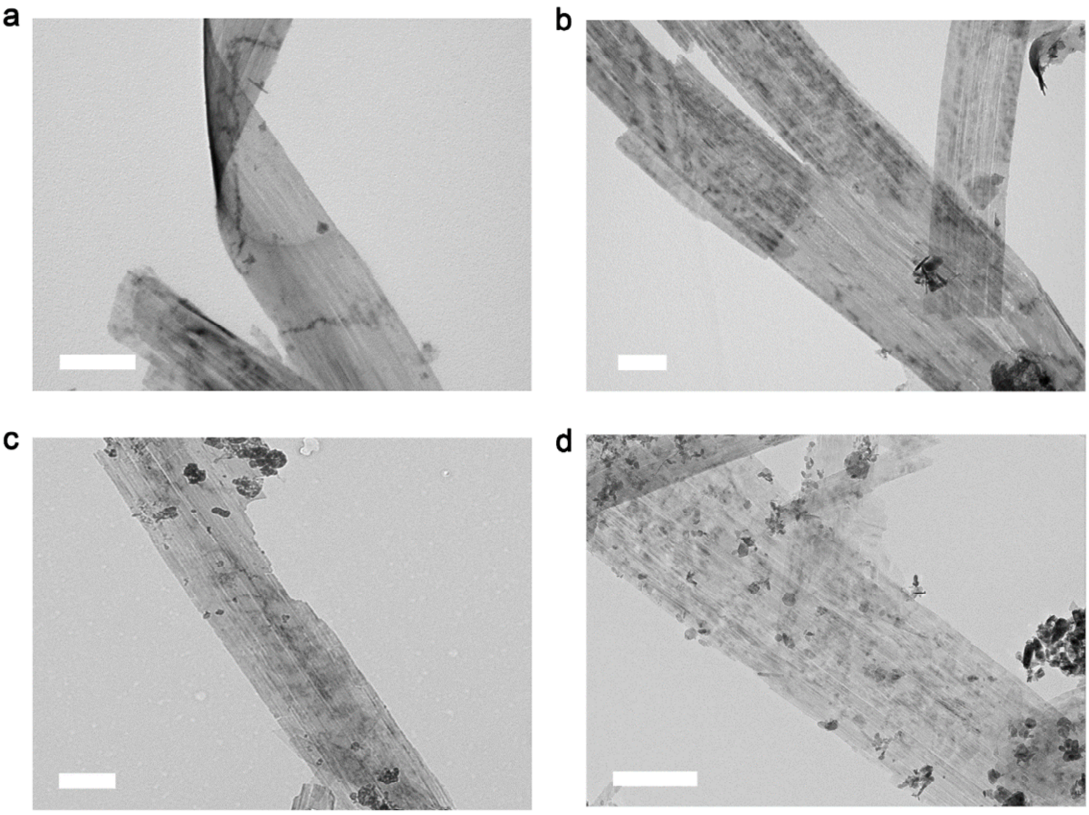

Supplementary Figure 11. TEM images. TEM images of the products achieved at 200 ^{o}C for various reaction times in 5 M KOH: **a,** 4 h, **b**, 8 h, **c,** 16 h and **d**, 20 h, respectively. Scale bars, 200 nm.

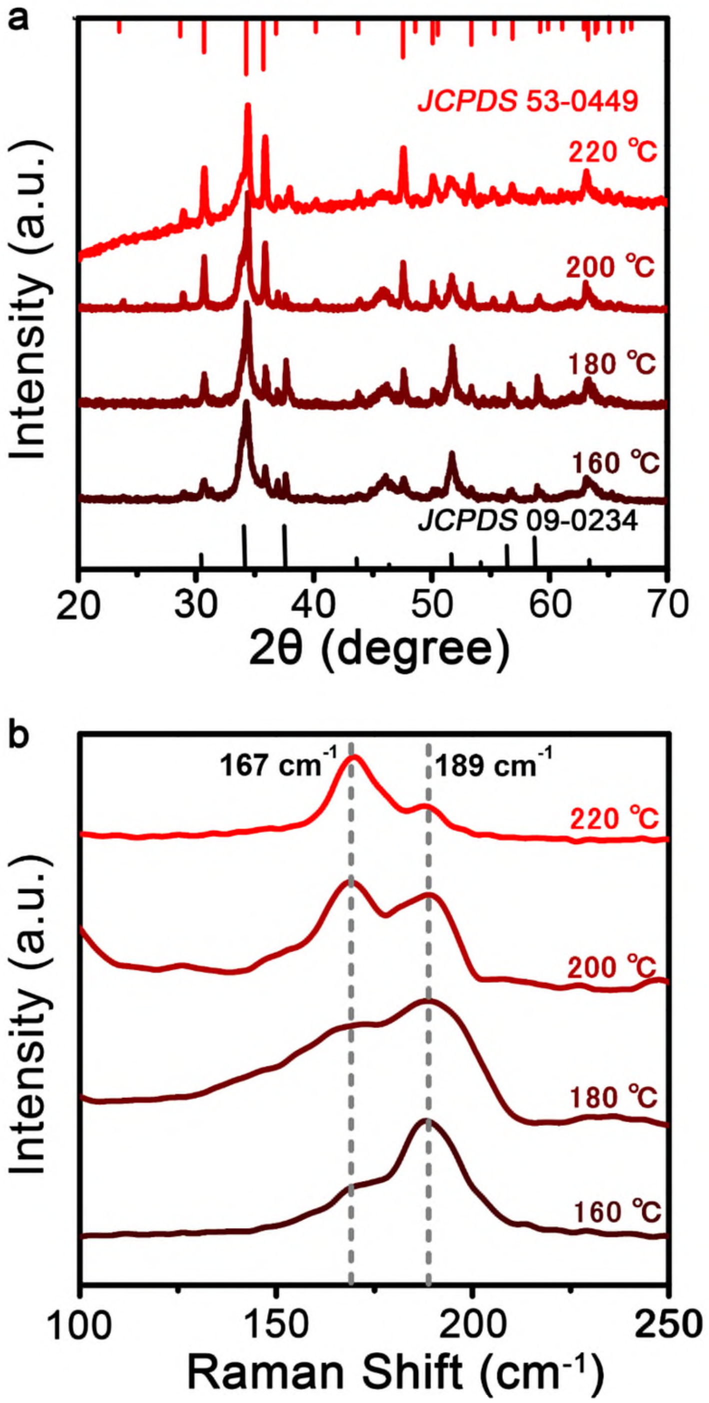

Supplementary Figure 12. XRD patterns and Raman spectra. XRD patterns (**a**) and Raman spectra (**b**) of the products achieved at various reaction temperatures for 12 h in 5 M KOH. The results reveal that the phase transition emerges at ~160 ℃ in 5 M KOH solution.

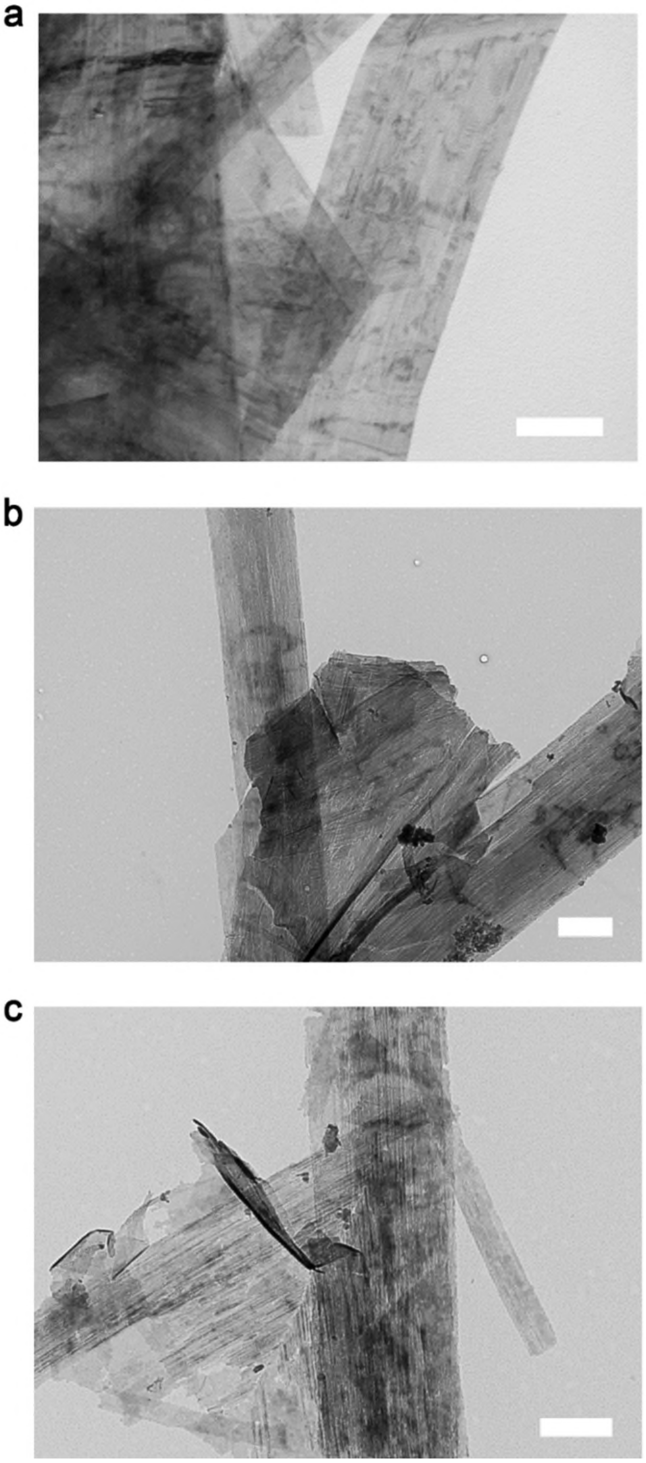

Supplementary Figure 13. TEM images. TEM images of the products achieved at various reaction temperatures for 12 h in 5 M KOH: **a,** 160 ^oC, **b,** 180 ^oC and **d,** 220 ^oC, respectively. Scale bars, 200 nm.

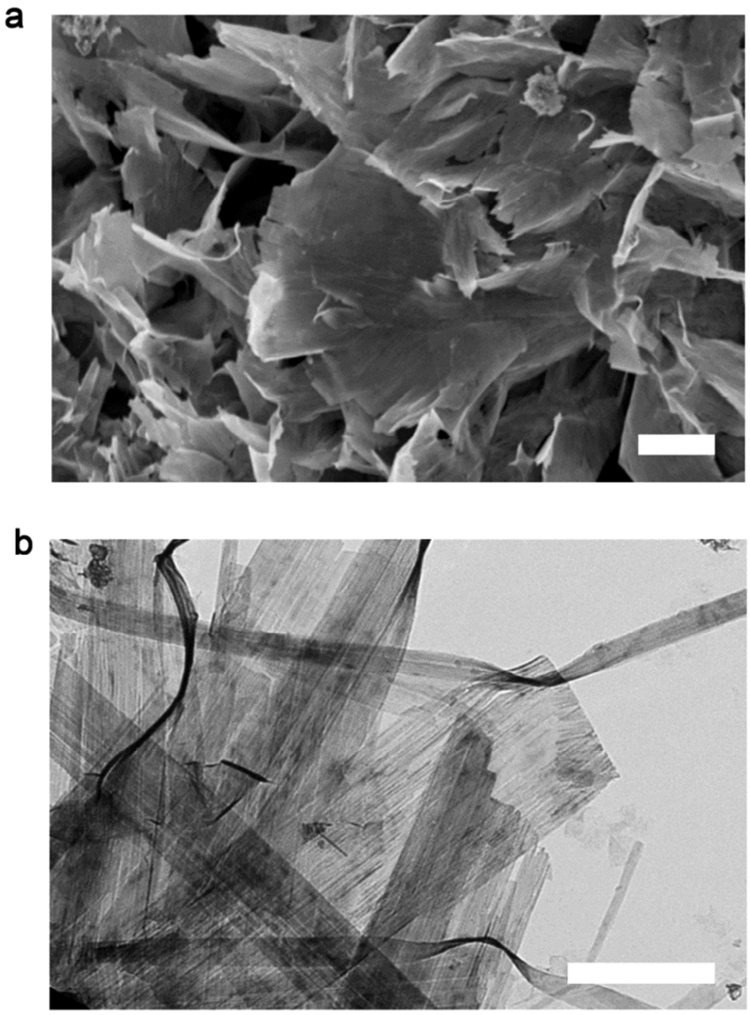

Supplementary Figure 14. The m-$CoSe_2$ nanobelts. SEM (**a**) and TEM (**b**) images of the sample obtained at 200 °C for 12 h in 5 M KOH, which reveal the m-$CoSe_2$ catalyst bears the porous structures. Scale bars, **a,** 1 μm and **b,** 500 nm.

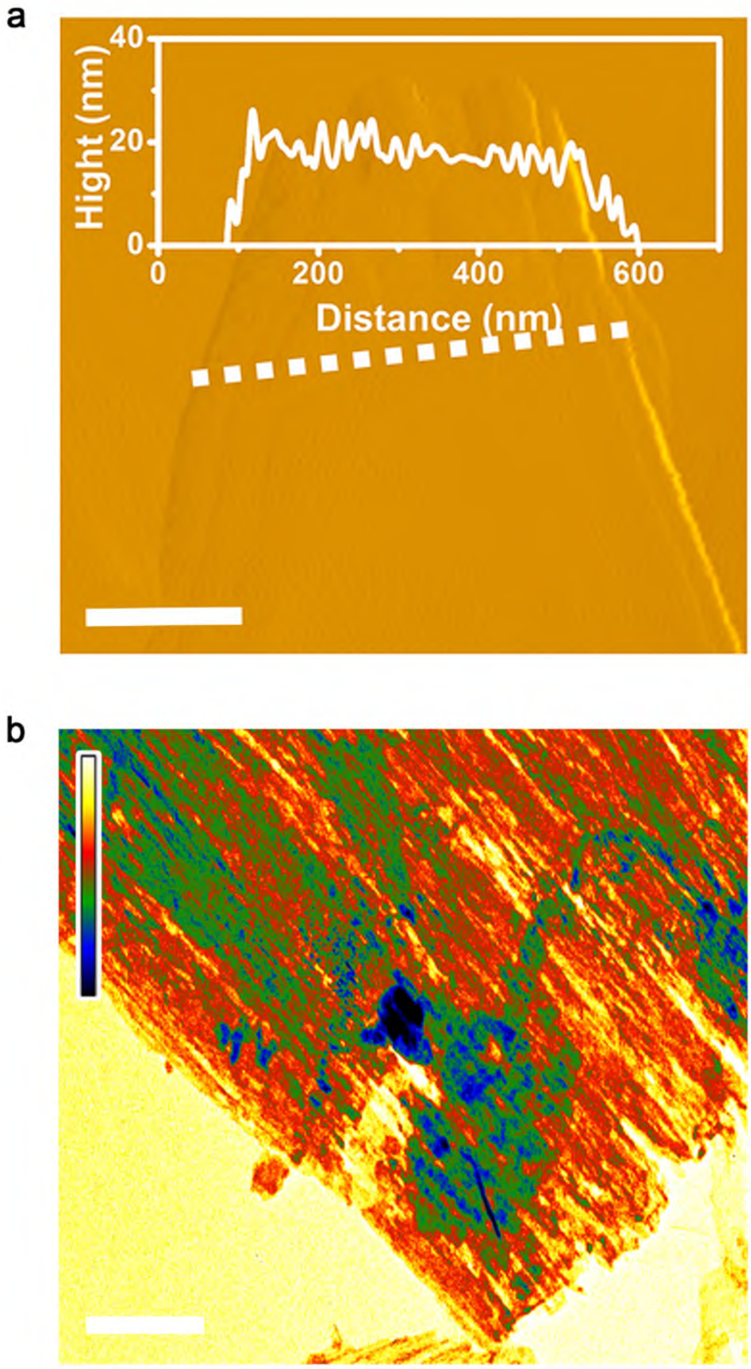

Supplementary Figure 15. Thickness measurements of m-$CoSe_2$. AFM image (**a**) and False-color HAADF image (**b**) of the m-$CoSe_2$. Scale bars, 200 nm. It clearly demonstrates plentifulslit nanopores on the surface of the m-$CoSe_2$.

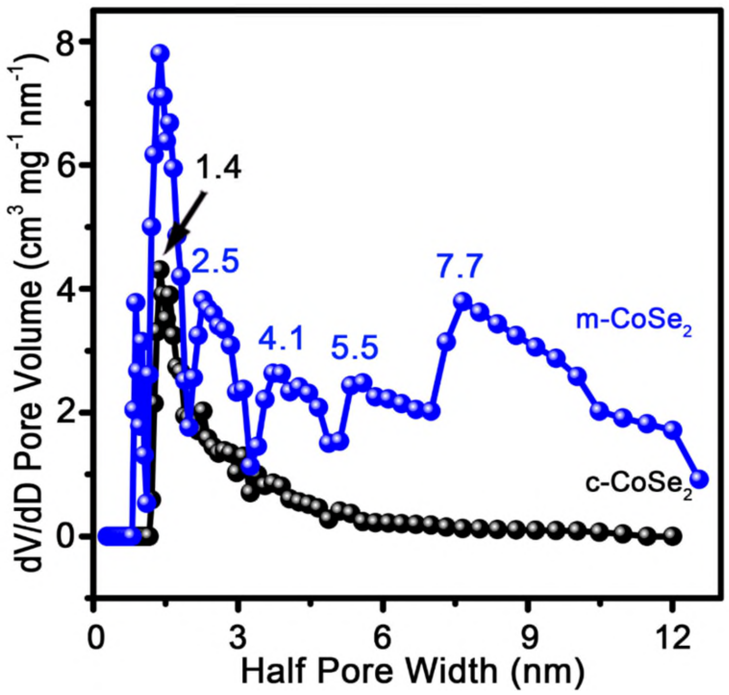

Supplementary Figure 16. The pore size distribution curves of c-$CoSe_2$ and m-$CoSe_2$, respectively. It shows that the m-$CoSe_2$ catalyst possesses a porous structure with size ranging from 1.4 to 7.7 nm. Such structure gives a high surface area, thus rich catalytic active sites for electrochemical reactions.

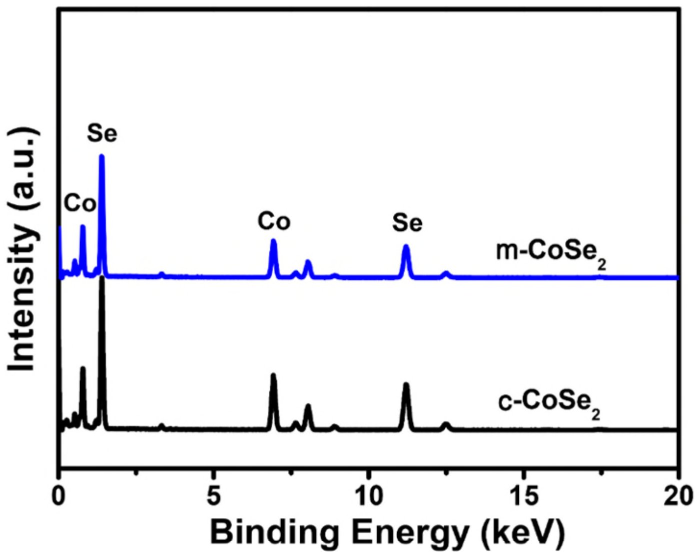

Supplementary Figure 17. EDX spectra. The EDX spectra of the m-$CoSe_2$ and c-$CoSe_2$, showing the ratio of the Co and Se of the m-$CoSe_2$ was almost consistent with fresh c-$CoSe_2$.

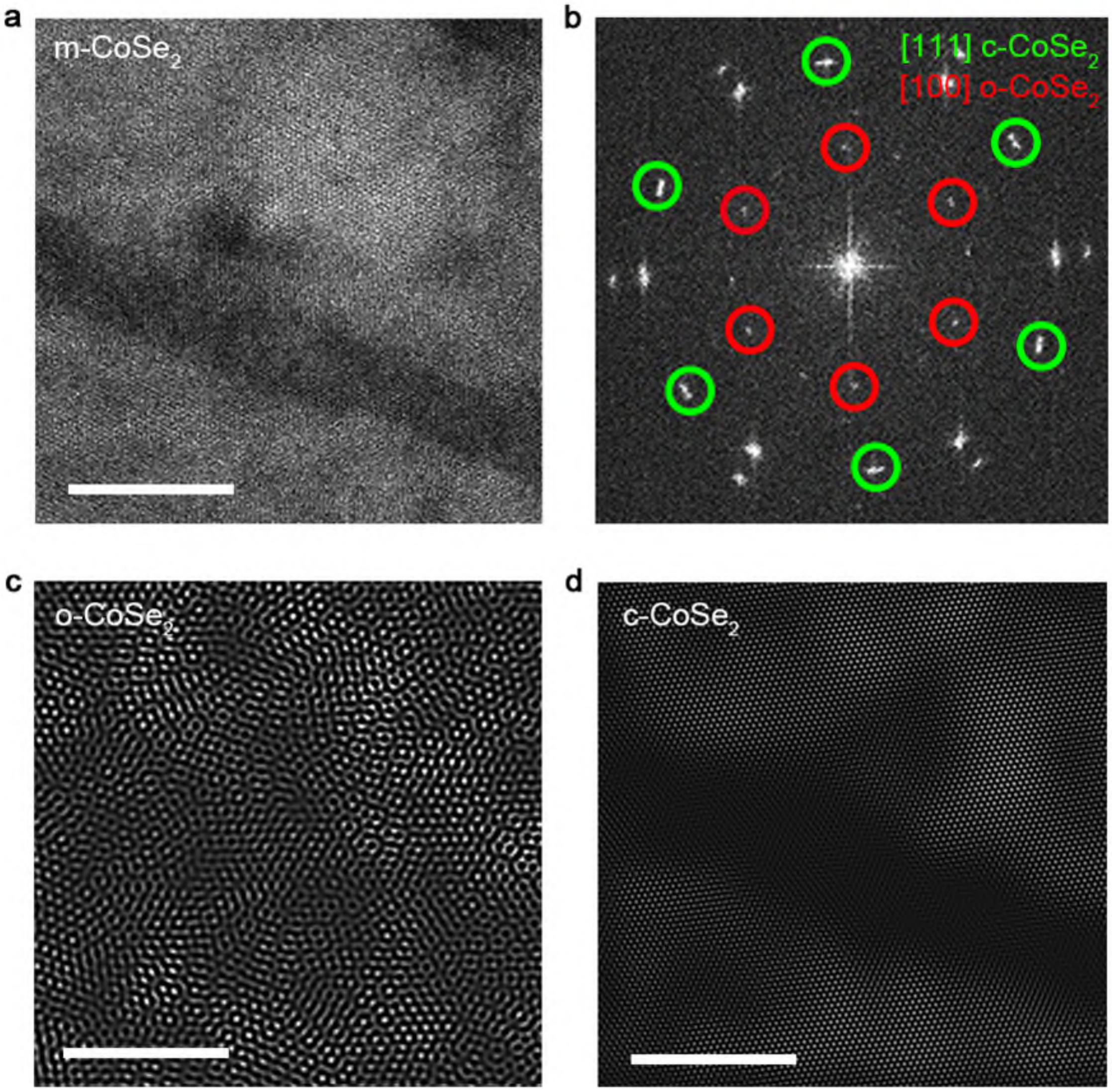

Supplementary Figure 18. FFT pattern. a, HAADF-STEM image of the m-$CoSe_2$. **b,** FFT pattern of the **a**. **c, d,** Inverse FFT patterns. Scale bars, 5 nm. Masking FFT pattern with only the o-$CoSe_2$ (red) or c-$CoSe_2$ (green) spots and then an inverse FFT is performed, an atomic-resolution image of o-$CoSe_2$ (**c**) or c-$CoSe_2$ (**d**) can be reconstructed. We colored these two inverse FFT patterns differently and overlap them (Figure 2c in Main-text), then the real-space atomic configuration can be reconstructed. Additionally, the two phases can also be straightforwardly revealed by the color-coded regions.

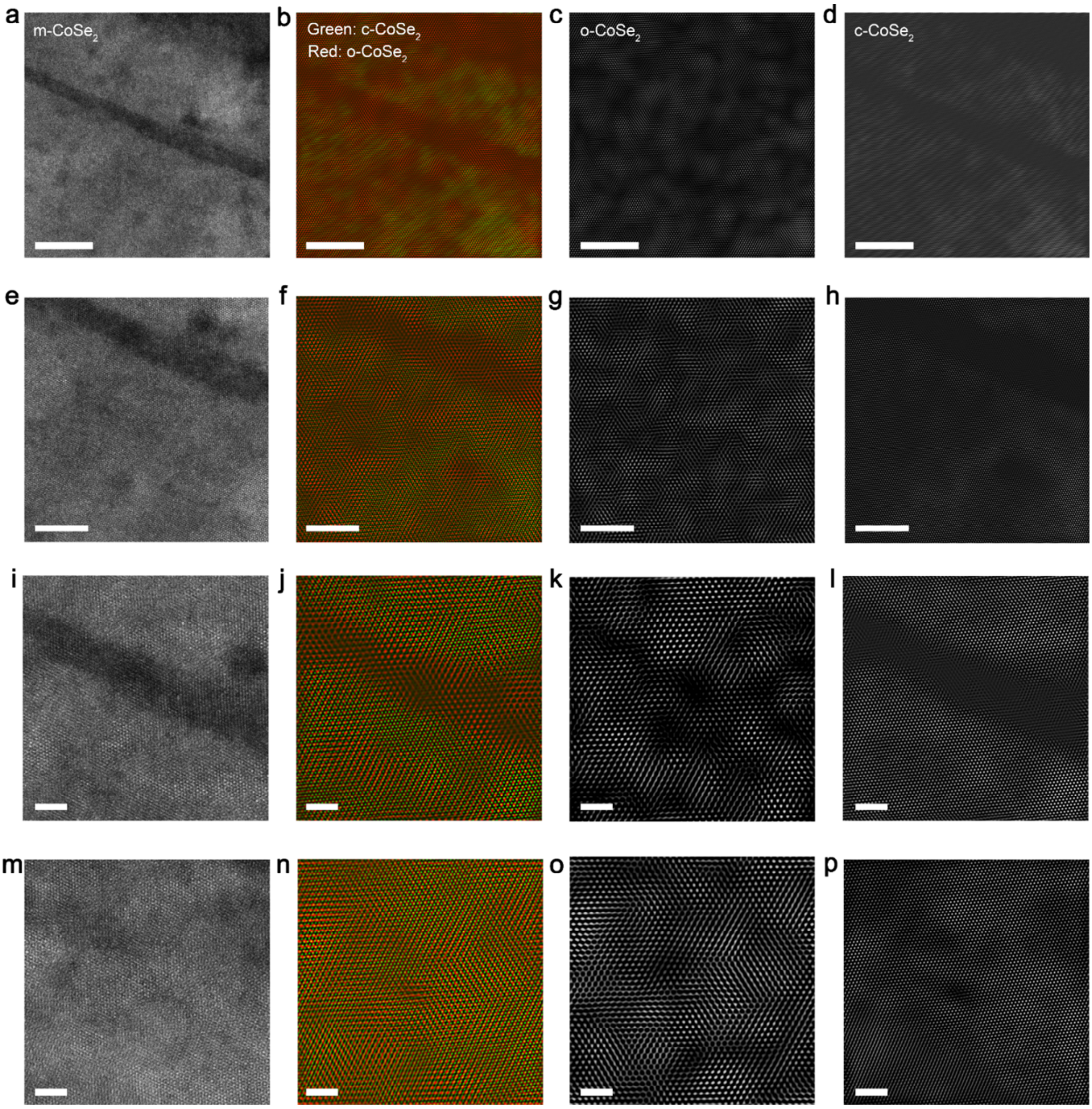

Supplementary Figure 19. HAADF and inverse FFT pattern of m-CoSe$_2$. a, e, i and **m,** HAADF images with different regions and magnifications show the high-crystalline of the as-prepared m-CoSe$_2$. **b, f, j** and **n,** Atomic-resolution image reconstructed by overlapping the inverse FFT patterns of **c** and **d**, **g** and **h**, **k** and **l**, **o** and **p**, respectively. The two kind of phase are color-coded in such a reconstructed image (red color: o-CoSe$_2$, green color: c-CoSe$_2$). **c, g, k** and **l,** Inverse spots belonging to o-CoSe$_2$ in FFT patterns, showing the real-space atomic configuration of o-CoSe$_2$. **d, h, I** and **p,** Atomic-resolution image reconstructed by inverse FFT pattern, masked spots of c-CoSe$_2$, showing atomic arrangement of the c-CoSe$_2$ in **a, e, i** and **m,** respectively. Sale bars in **a-d**: 10 nm; Sale bars in **e-h**: 5 nm; Sale bars in **i-p**: 2 nm.

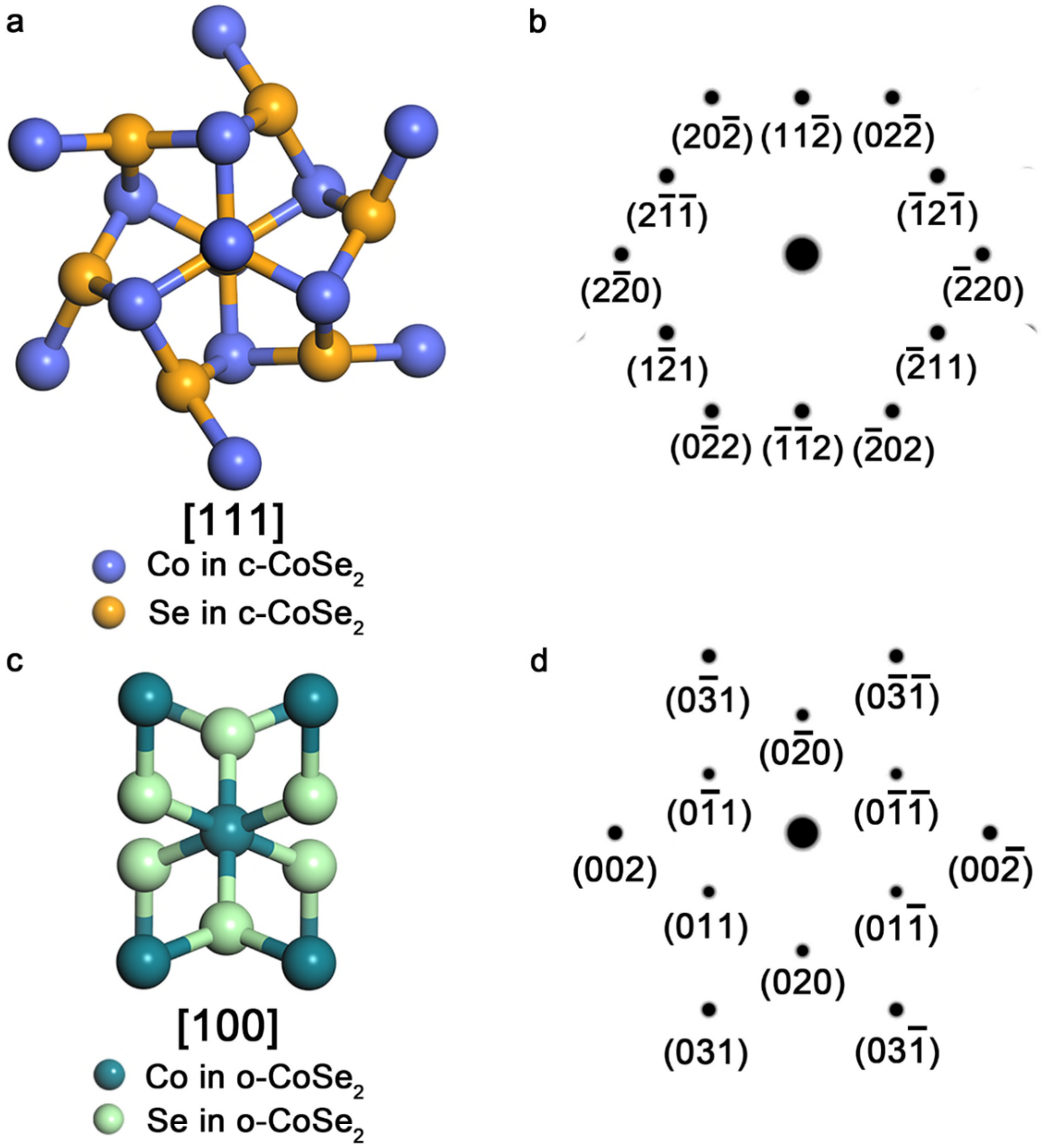

Supplementary Figure 20. Ball-and-stick model. a, b, Ball-and-stick model of the c-$CoSe_2$ (zone axis: [111]) and its corresponding simulated FFT pattern, which was consistent with FFT pattern demonstrated in Figure 2e. **c, d,** Ball-and-stick model of the o-$CoSe_2$ (zone axis: [100]) and its corresponding simulated FFT pattern, which was same as the FFT pattern demonstrated in Figure 2f.

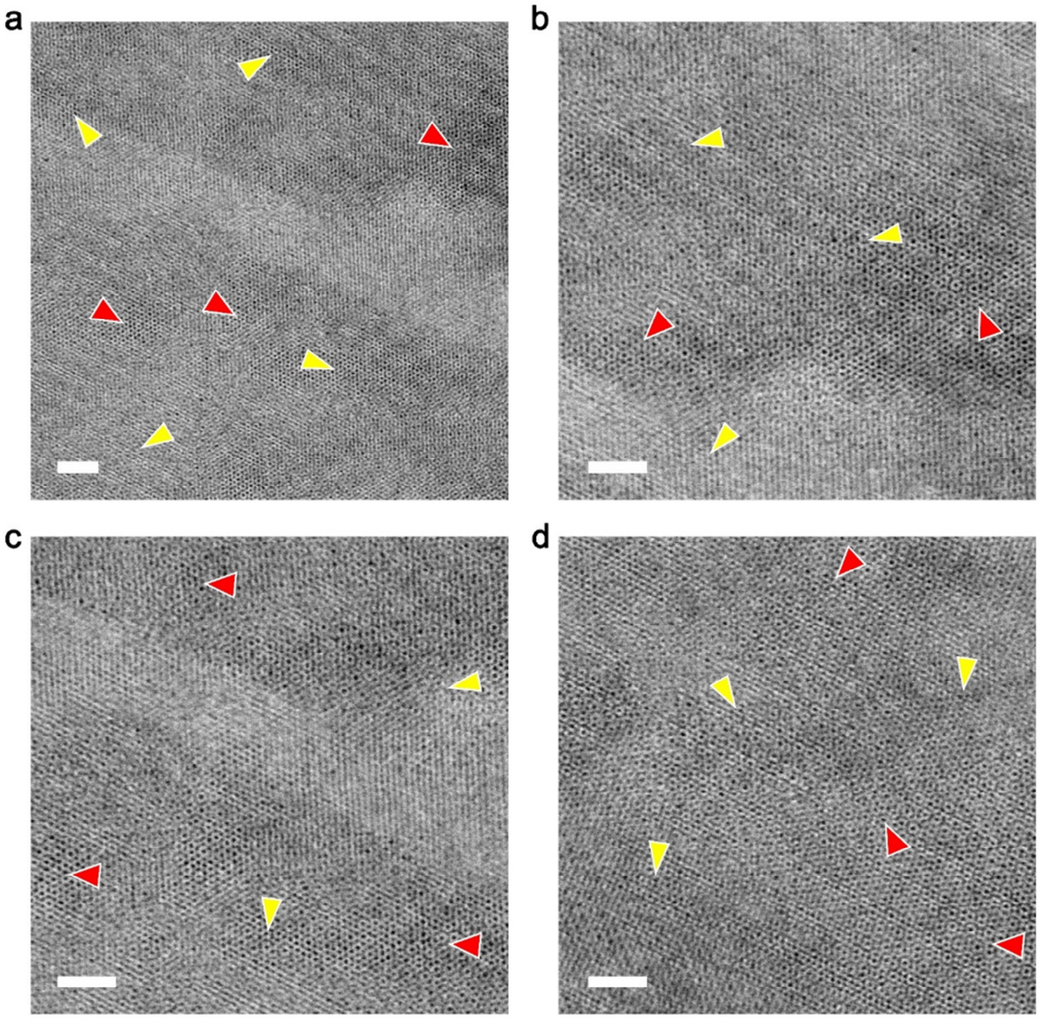

Supplementary Figure 21. High-resolution TEM (HRTEM) images of m-$CoSe_2$. a-d, HRTEM images of m-$CoSe_2$ at different regions, showing a high-crystalline structure with nearly homogeneous distribution of cubic and orthorhombic phases. Red arrows refer to cubic structure and yellow arrows refer to orthorhombic structure. Scale bars, 2 nm.

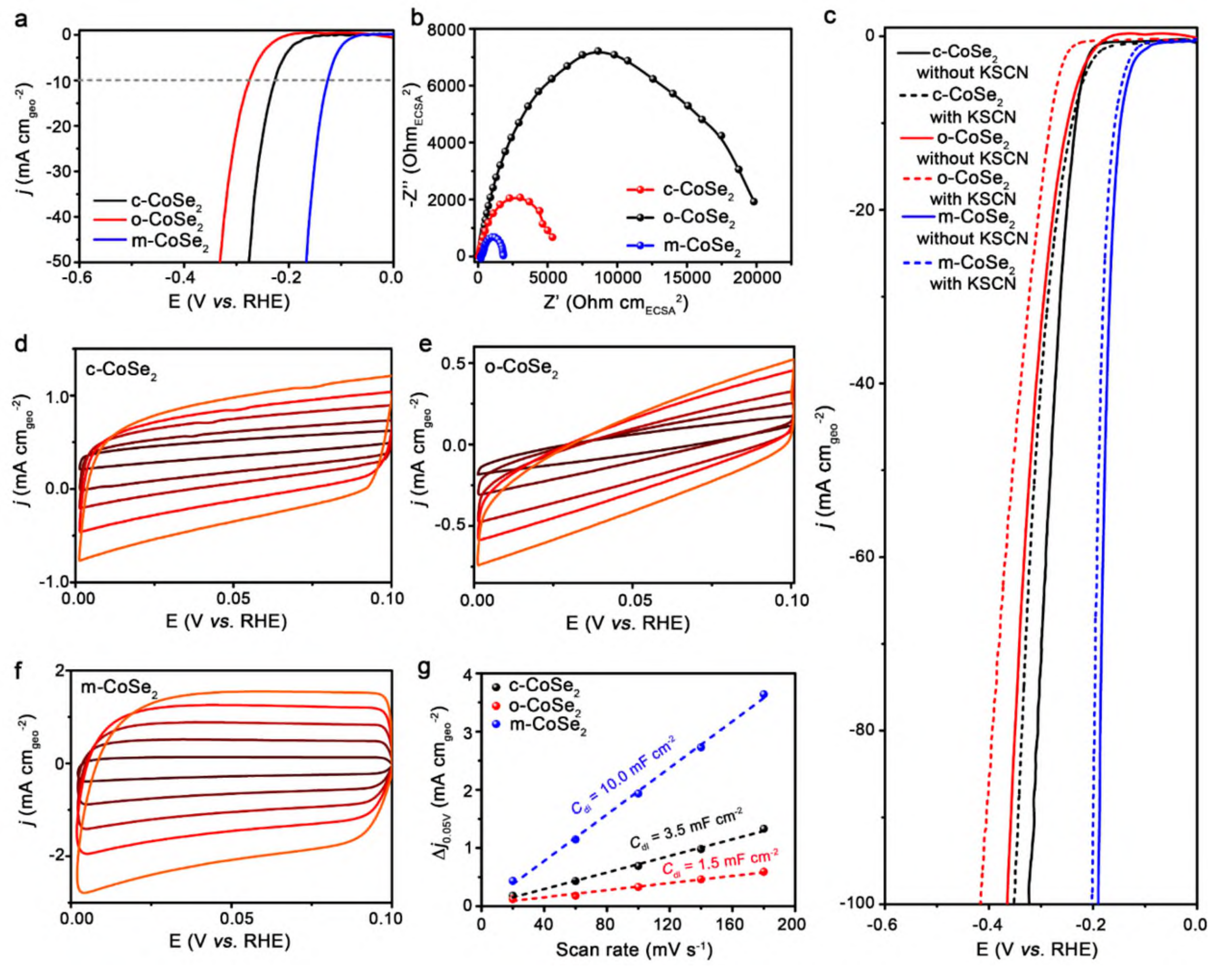

Supplementary Figure 22. HER properties in 0.5 M H_2SO_4. a, HER polarization curves of m-, c- and o-$CoSe_2$ catalysts. Catalyst loading: ~1.02 mg cm^{-2}. Sweep rate: 2 mV s^{-1}. **b,** EIS Nyquist plots of m-, c- and o-$CoSe_2$ catalysts. Plots showing the extraction of the C_{dl} for various catalysts. **c,** The influence of SCN^- on the HER activity of investigated catalysts was evaluated by adding 10 mM SCN^- in the electrolyte. Compared with c- and o-$CoSe_2$ whose activity were degraded greatly, the negligible HER deactivation for m-$CoSe_2$ evidences that such a porous structure can provide more active sites. **d-f,** CV in the region of 0.0~0.1 V *vs*. RHE for c-, o- and m-$CoSe_2$ catalysts. **g,** Plots showing the extraction of the C_{dl} for various catalysts**.** Double-layer capacitance (C_{dl}), which scales roughly with the effective electrochemically active surface area. Our results reveal a considerably larger C_{dl} of m-$CoSe_2$ (10.0 mFcm^{-2}) compared with c-$CoSe_2$ (3.5 mFcm^{-2}) and o-$CoSe_2$ (1.5 mFcm^{-2}), suggesting more accessible active sites created on m-$CoSe_2$ catalyst.

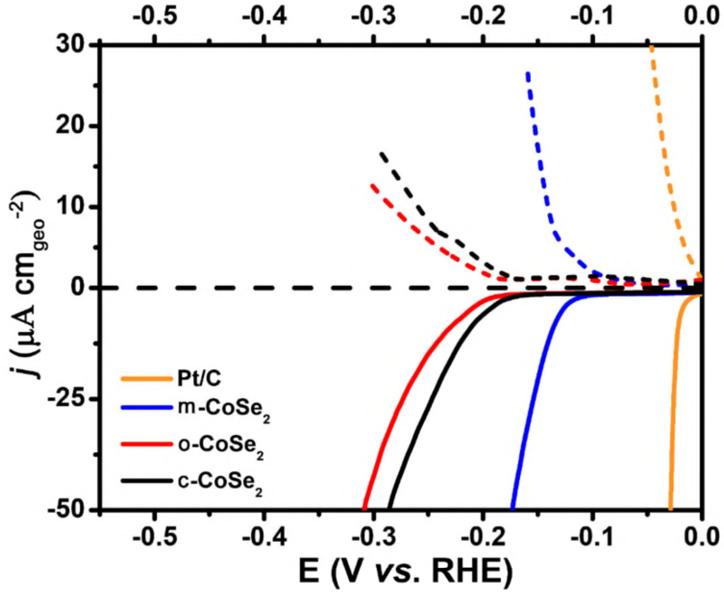

Supplementary Figure 23. Rotating ring disk electrode tests. An RRDE with both a glassy carbon disk (5.61 mm diameter, disk area: 0.2475 cm^2) and a Pt ring (6.25 mm inner-diameter and 7.92 mm outer diameter, ring area: 0.1866 cm^2) was used for confirming the H_2 evolution. The Pt-ring electrode was kept at 0.5 V for the oxidation of H_2 that was generated on the disk electrode, which verify that hydrogen oxidation reaction (HOR) happen on Pt ring, revealing the production of H_2 under the test condition.

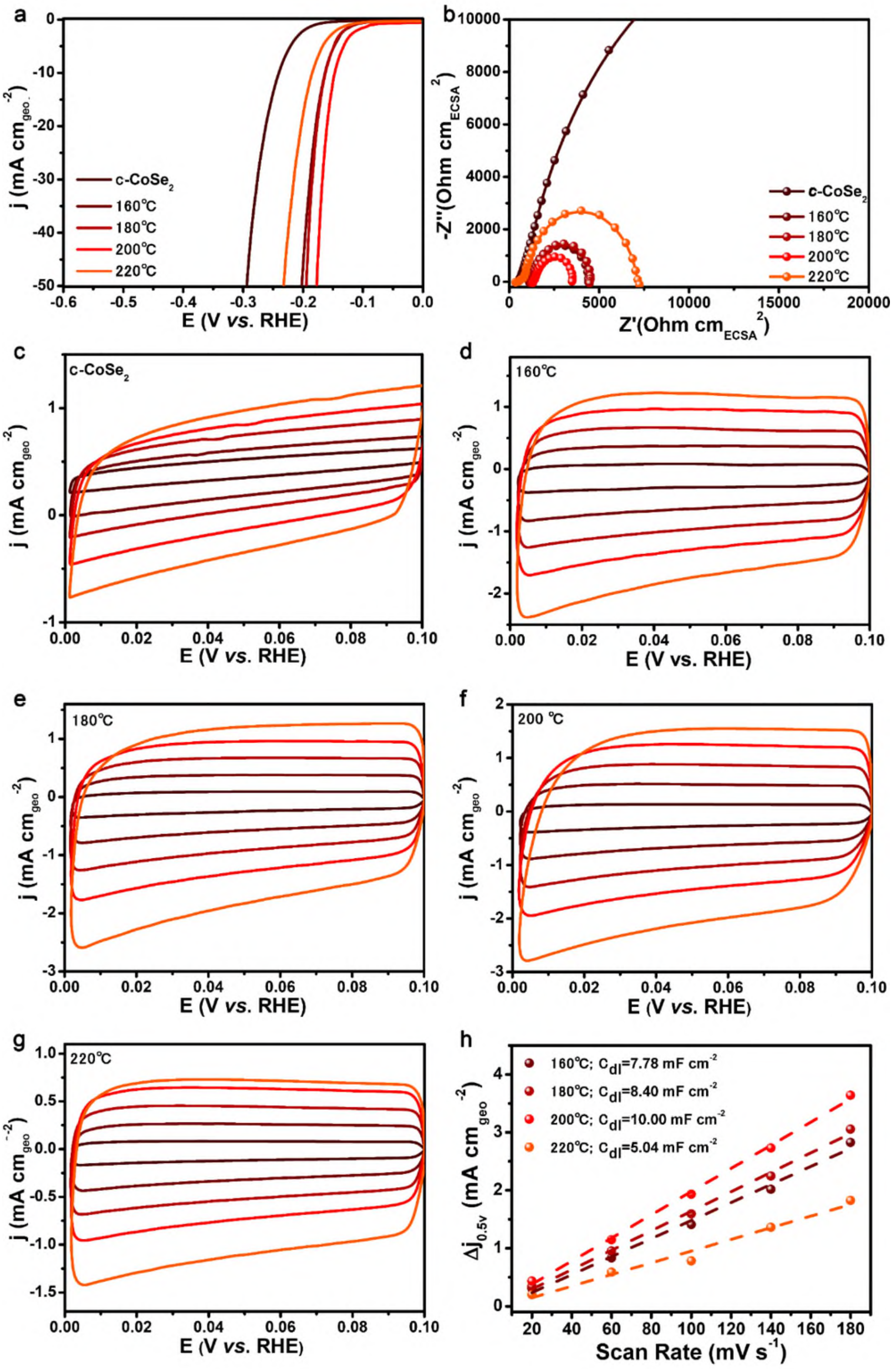

Supplementary Figure 24. Influence of temperature on the HER properties in 0.5 M H_2SO_4. a, HER polarizaiton curves, **b,** EIS Nyquist plots, **d-h,** CV in the region of 0.0~0.1 V vs. RHE for c- and m-$CoSe_2$ prepared at various temperature. **h,** Plots showing the extraction of the C_{dl}. It reveals that the optimized m-$CoSe_2$ catalyst is achieved at 200 ^oC.

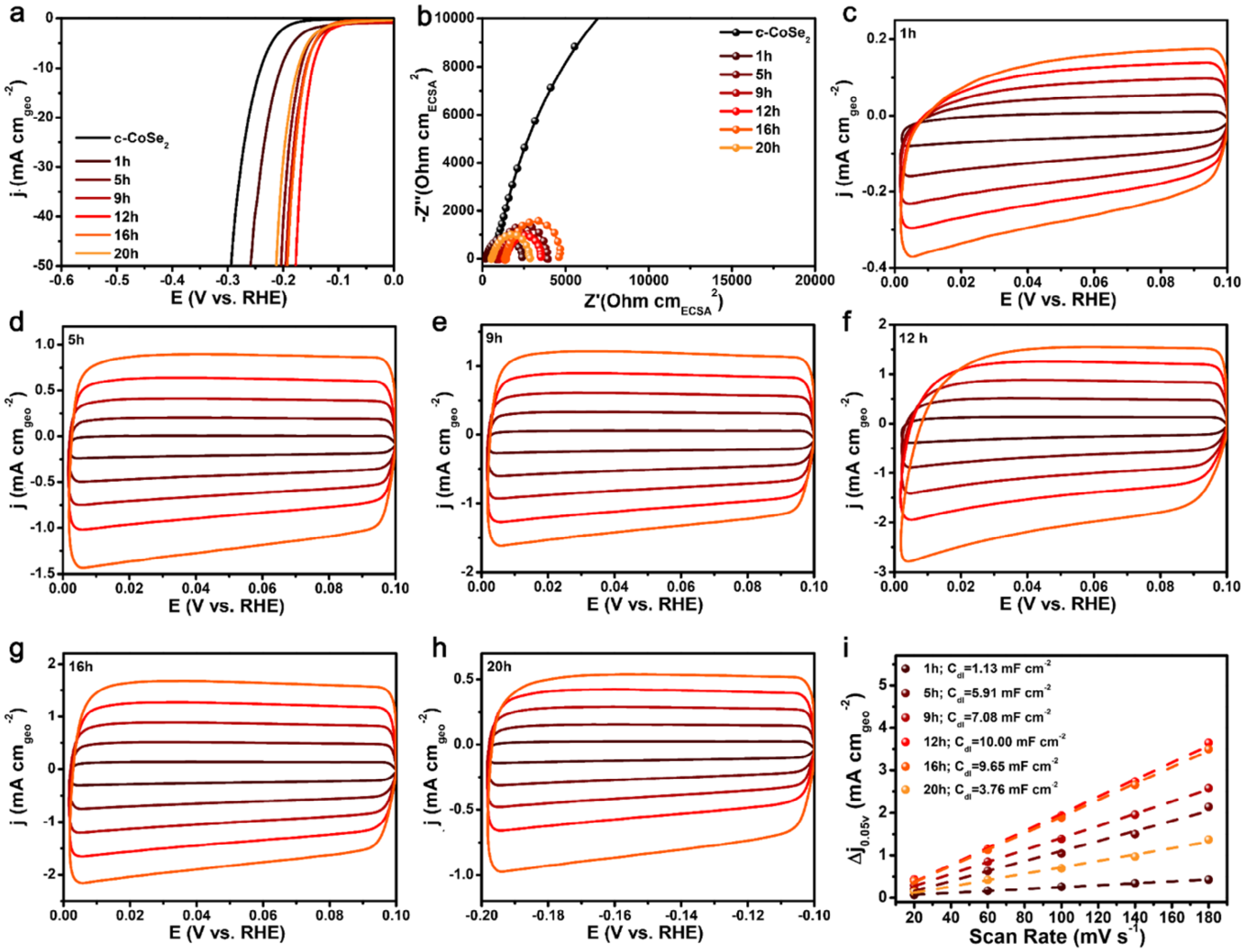

Supplementary Figure 25. Influence of reaction time on the HER properties in 0.5 M H_2SO_4. a, HER polarizaiton curves, **b,** EIS Nyquist plots, **c-h,** CV in the region of 0.0~0.1 V vs. RHE. **i,** Plots showing the extraction of the C_{dl} for c- and m-$CoSe_2$ prepared at various temperature. It reveals that the optimized m-$CoSe_2$ catalyst is achieved at 12 h.

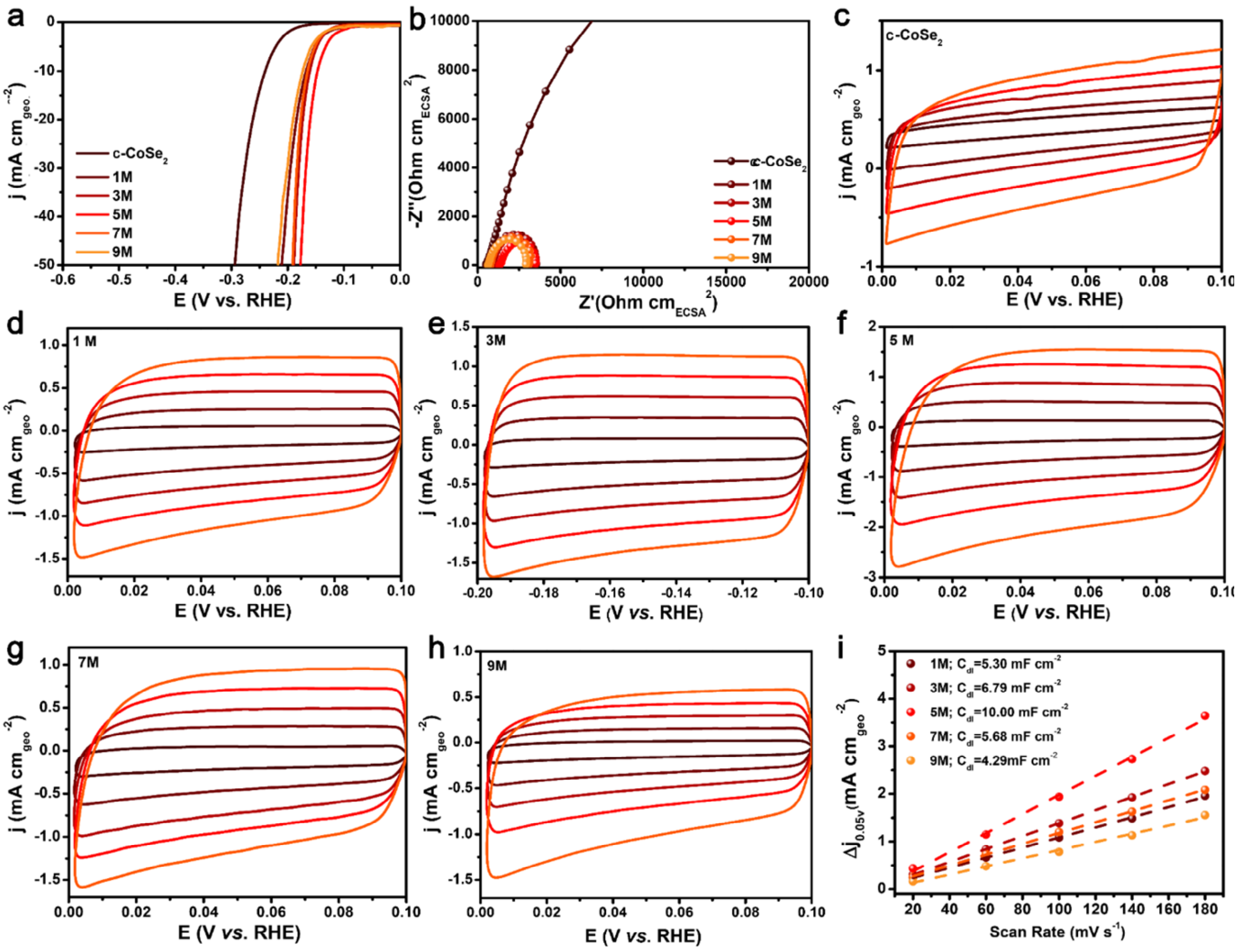

Supplementary Figure 26. Influence of KOH concentration on the HER properties in 0.5 M H_2SO_4. a, HER polarizaiton curves, **b,** EIS Nyquist plots, **c-h,** CV in the region of 0.0~0.1 V *vs.* RHE for c- and m-$CoSe_2$ prepared at different KOH concentration. **i,** Plots showing the extraction of the C_{dl}. It reveals that the optimized m-$CoSe_2$ catalyst is obtained at 5 M KOH.

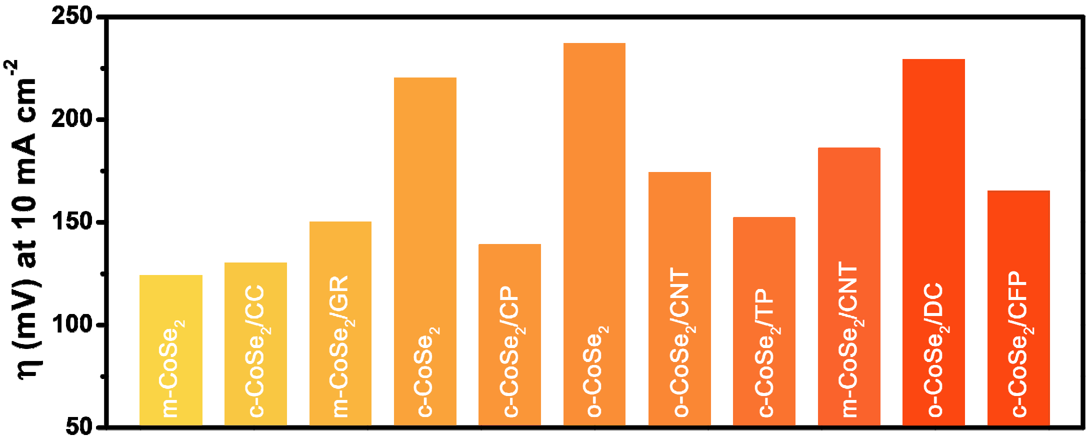

Supplementary Figure 27. Comparison of catalytic parameters of m-$CoSe_2$ and other $CoSe_2$ catalysts in 0.5 M H_2SO_4. c-$CoSe_2$/CC (Ref. 1), m-$CoSe_2$/GR (Ref. 2), c-$CoSe_2$ (Ref. 3), c-$CoSe_2$/CP (Ref. 4), o-$CoSe_2$ (Ref. 5), o-$CoSe_2$/CNT (Ref. 6), c-$CoSe_2$/TP (Ref. 7), m-$CoSe_2$/CNT (Ref. 8), o-$CoSe_2$/DC (Ref. 9), c-$CoSe_2$/CFP (Ref. 10). CC = Carbon cloth; GR = Graphene; CP = Carbon paper; CNT = Carbon nanotube; TP = Ti Plate; DC = Defective Carbon; CFP = Carbon fiber paper;

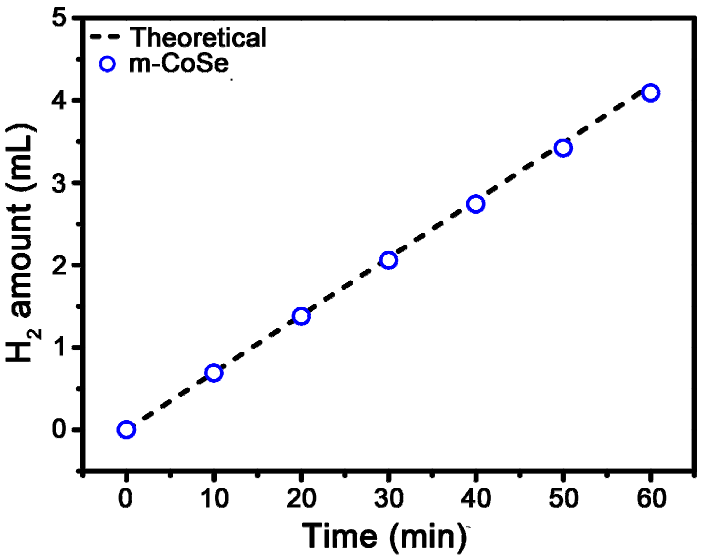

Supplementary Figure 28. Current efficiency for H_2 evolution catalyzed by the m-CoSe$_2$ electrode. It shows that the yields of H_2 analyzed by gas chromatography. The detected amounts of H_2 agree well with the theoretical values, indicating a device Faradaic efficiency of 99.6%.

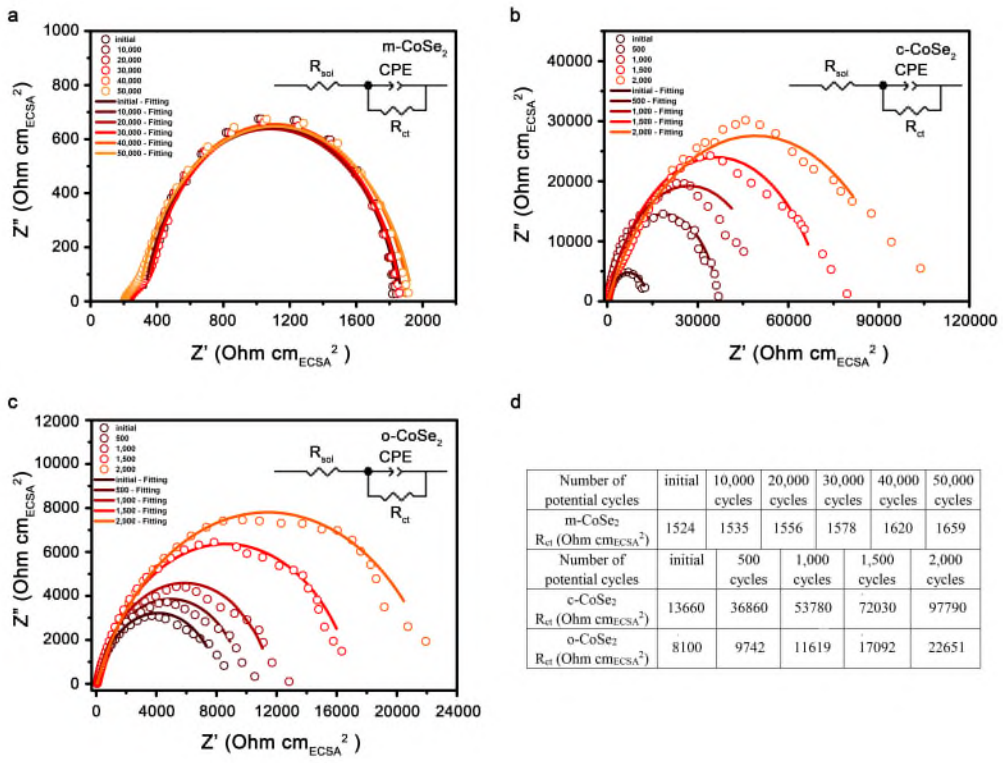

Number of potential cycles	initial	10,000 cycles	20,000 cycles	30,000 cycles	40,000 cycles	50,000 cycles
m-CoSe2 R_{ct} (Ohm cm_{ECSA}^2)	1524	1535	1556	1578	1620	1659
Number of potential cycles	initial	500 cycles	1,000 cycles	1,500 cycles	2,000 cycles	
c-CoSe2 R_{ct} (Ohm cm_{ECSA}^2)	13660	36860	53780	72030	97790	
o-CoSe2 R_{ct} (Ohm cm_{ECSA}^2)	8100	9742	11619	17092	22651	

Supplementary Figure 29. EIS Nyquist plots. a-c, EIS Nyquist plots of the m-, c- and o-$CoSe_2$ catalysts before and after different potential cycles, respectively (Insert: R(QR) equivalent circuit for Nyquist plots). **d,** The R(QR) equivalent circuit was used to fit EIS results. The constant phase element (CPE) is used to replace the pure capacitance due to the non-ideal capacitive response. As can be seen from **a-c**, the EIS data matches well with the fitting curves.

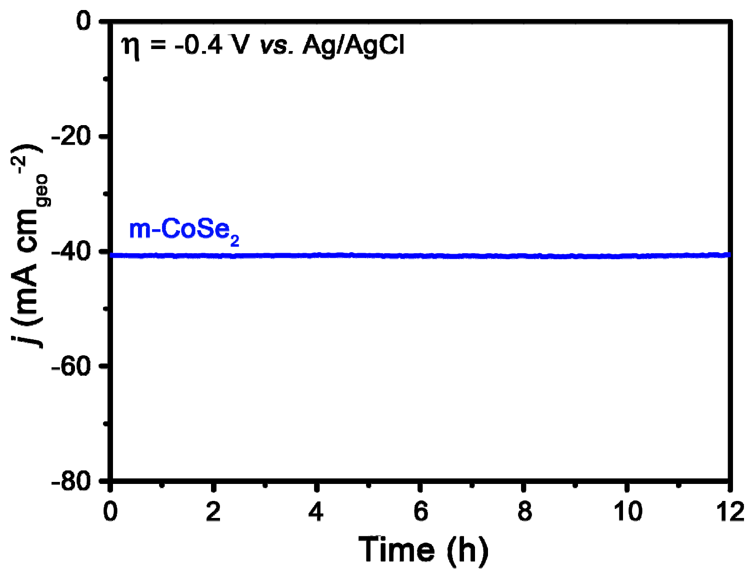

Supplementary Figure 30. Electrochemical stability of m-$CoSe_2$. We performed aggressive long-term stability tests on m-$CoSe_2$ catalyst by means of chronoamperometry (*j* ~ t), showing no current decay over 12 h of continuous operation at -400 mV *vs*. Ag/AgCl in 0.5 M H_2SO_4.

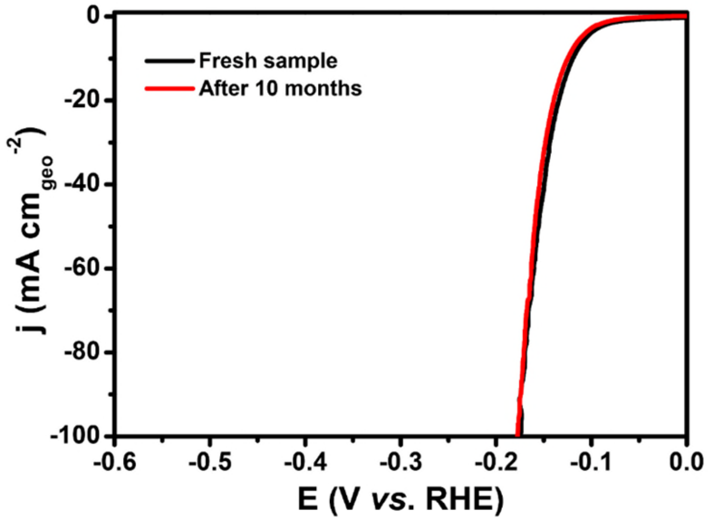

Supplementary Figure 31. Storage stability. Polarization curves (with iR-corrected) recorded from m-$CoSe_2$ before and after 10 months of storage under lab environment (stored in an airtight sample tube), revealing that no obvious degradation of HER activity was detected.

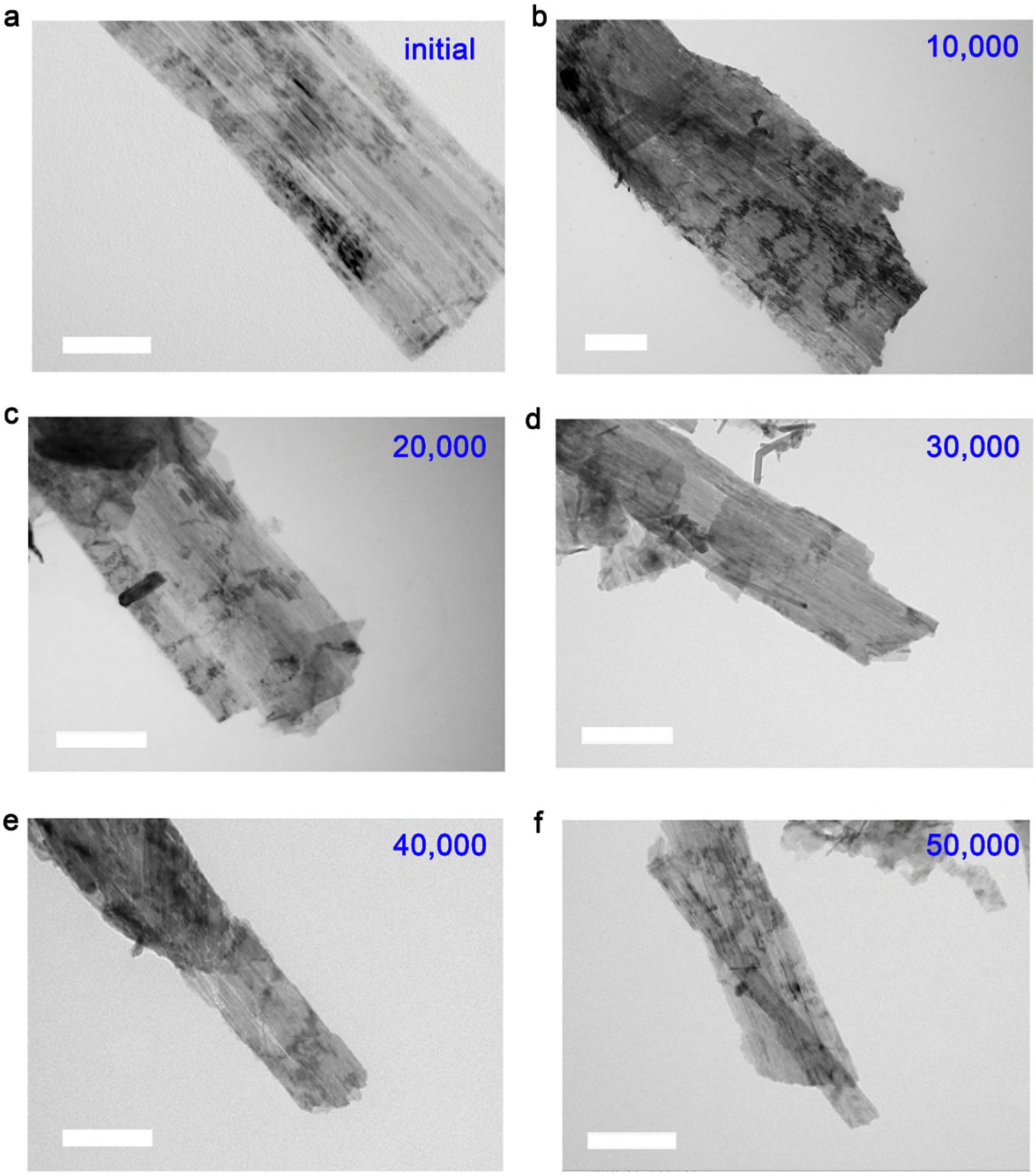

Supplementary Figure 32. TEM images. a-f, TEM images of the m-$CoSe_2$ before and after 10,000, 20,000, 30,000, 40,000 and 50,000 cycles, which show that the m-$CoSe_2$ can retain the original morphology after stability test. Scale bars, 200 nm.

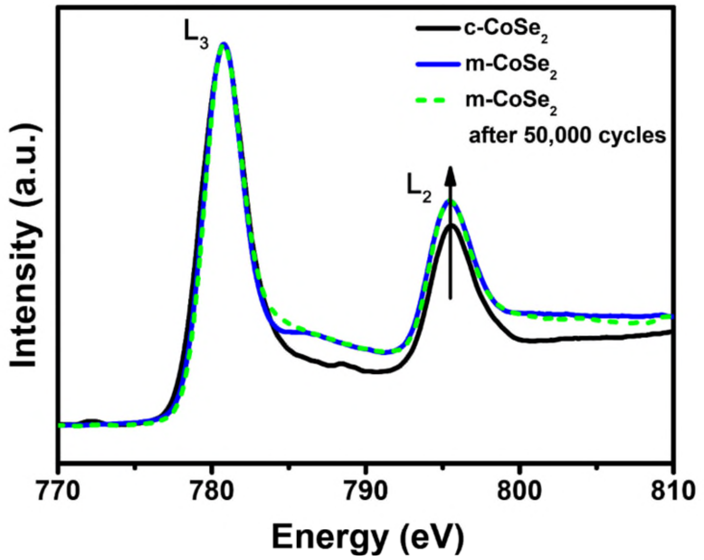

Supplementary Figure 33. Co L-edge measurements. $L_{2,3}$ edges of Co for m- and c-$CoSe_2$. Compared to c-$CoSe_2$, the Co L_2 edge of the m-$CoSe_2$ shows the increasing intensity, suggesting an increase in the Co oxidation state and a decreased occupancy of the antibonding e_g* orbitals. By comparing the Co L-edge of m-$CoSe_2$ before and after 50,000 potential cycles, the conclusion that electronic structure of m-$CoSe_2$ was not perturbed can be summarized.

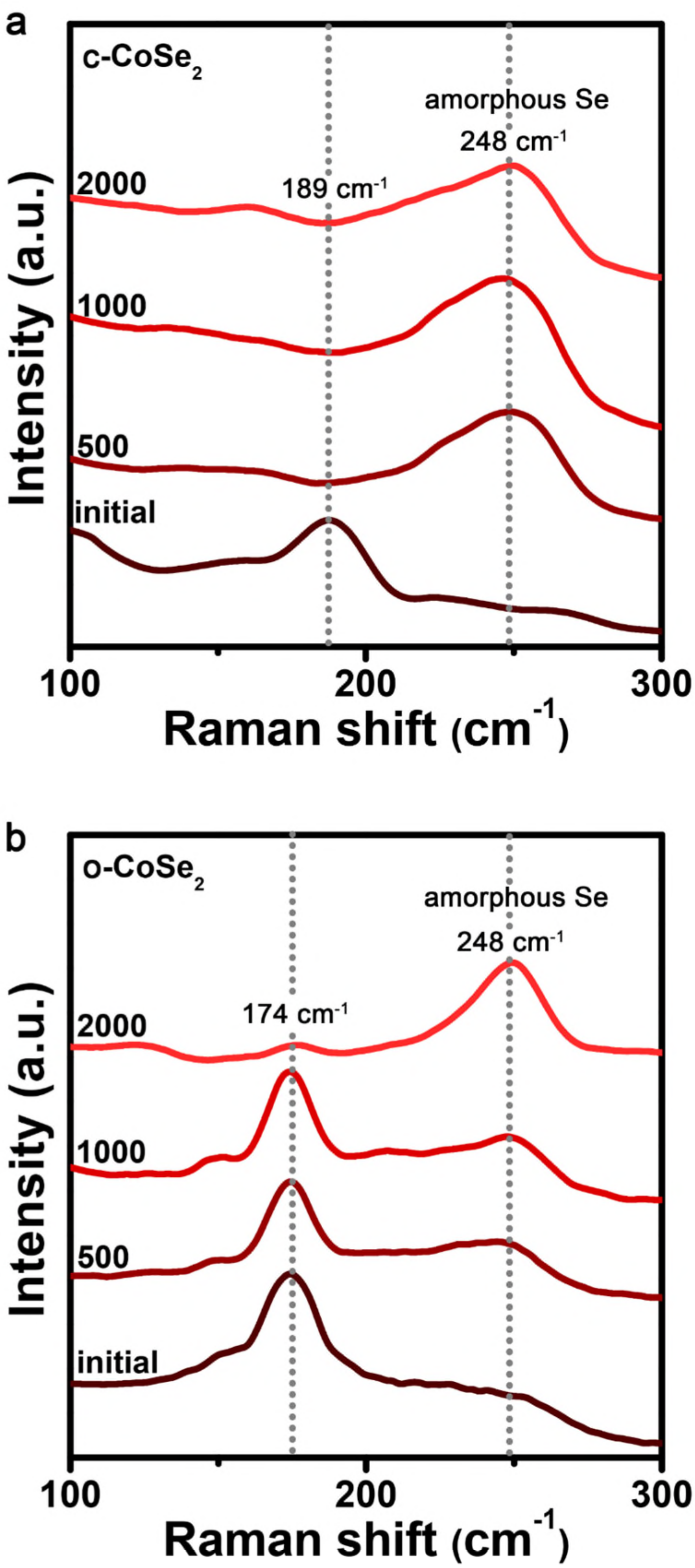

Supplementary Figure 34. Raman spectra. a, **b,** Raman spectra of the c- and o-$CoSe_2$ before and after 500, 1,000 and 2,000 cycles, respectively. The Raman spectra display new active peak at 248 cm^{-1} resulted from amorphous Se, indicating the structural degradation of c- and o-$CoSe_2$.

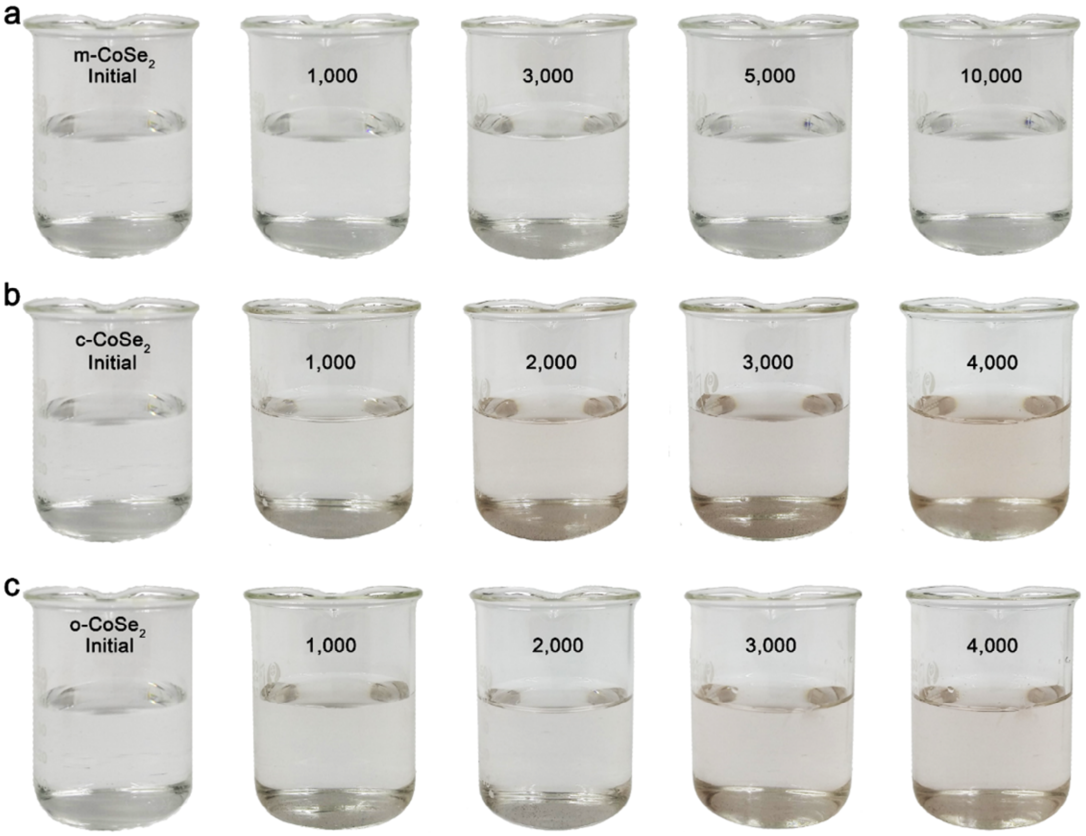

Supplementary Figure 35. Colorimetric comparison experiment. a-c, Optical images show cycled electrolytes of the m-, c- and o-$CoSe_2$ catalysts, respectively. These results reveal that a light red color was observed for c-$CoSe_2$ and o-$CoSe_2$ since the Se leaching, while almost no color change was observed for m-$CoSe_2$, indicating its good structural stability.

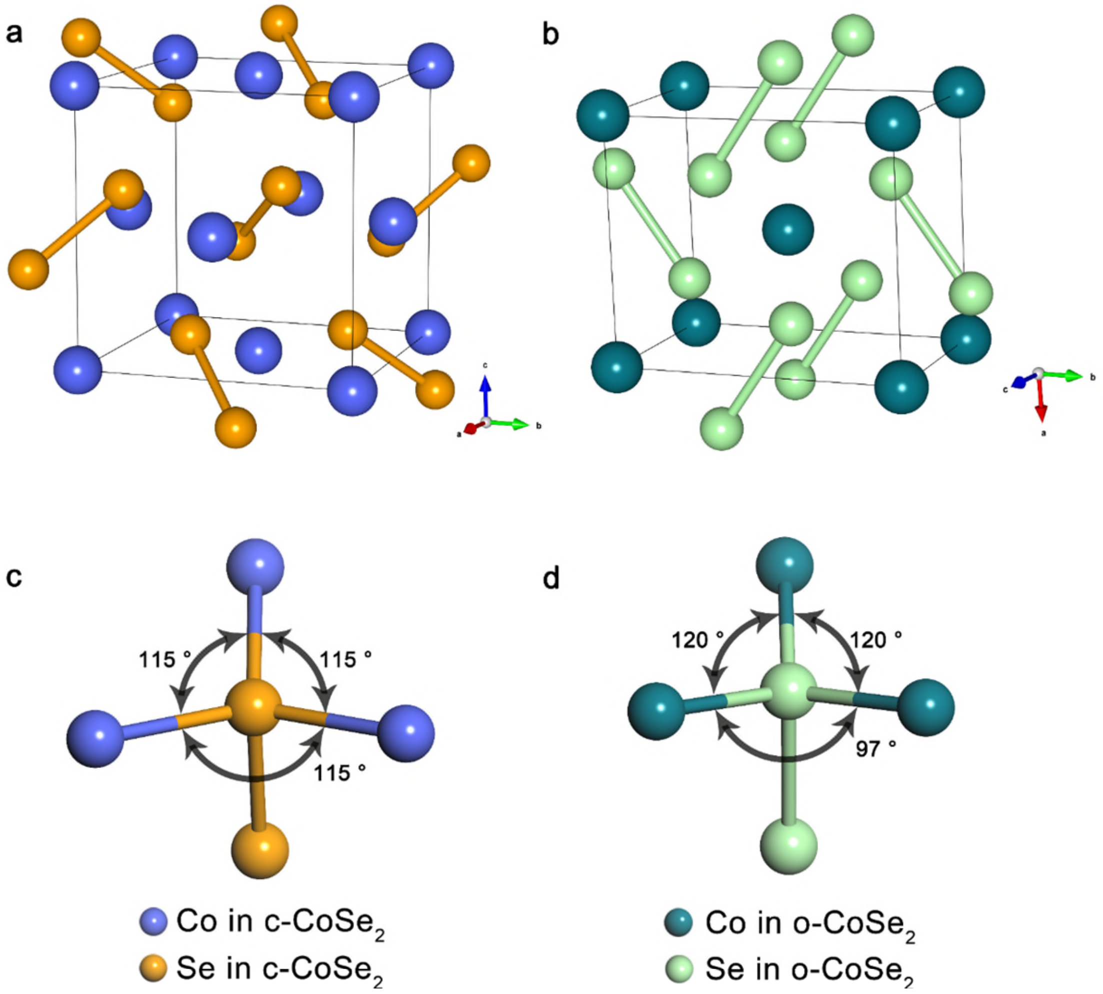

Supplementary Figure 36. Crystal structure. a, b, Crystal structure of the c- and o-$CoSe_2$, respectively, which show that the both phases bear the characteristic feature of covalently bonded Se-Se pairs**. c, d,** The structure of the Se-Se pair in c- and o-$CoSe_2$, respectively. c-$CoSe_2$ possesses three Co-Se-Co angles of 115 °and o-$CoSe_2$ contains one Co-Se-Co angle of 97 ° and two of 120 °.

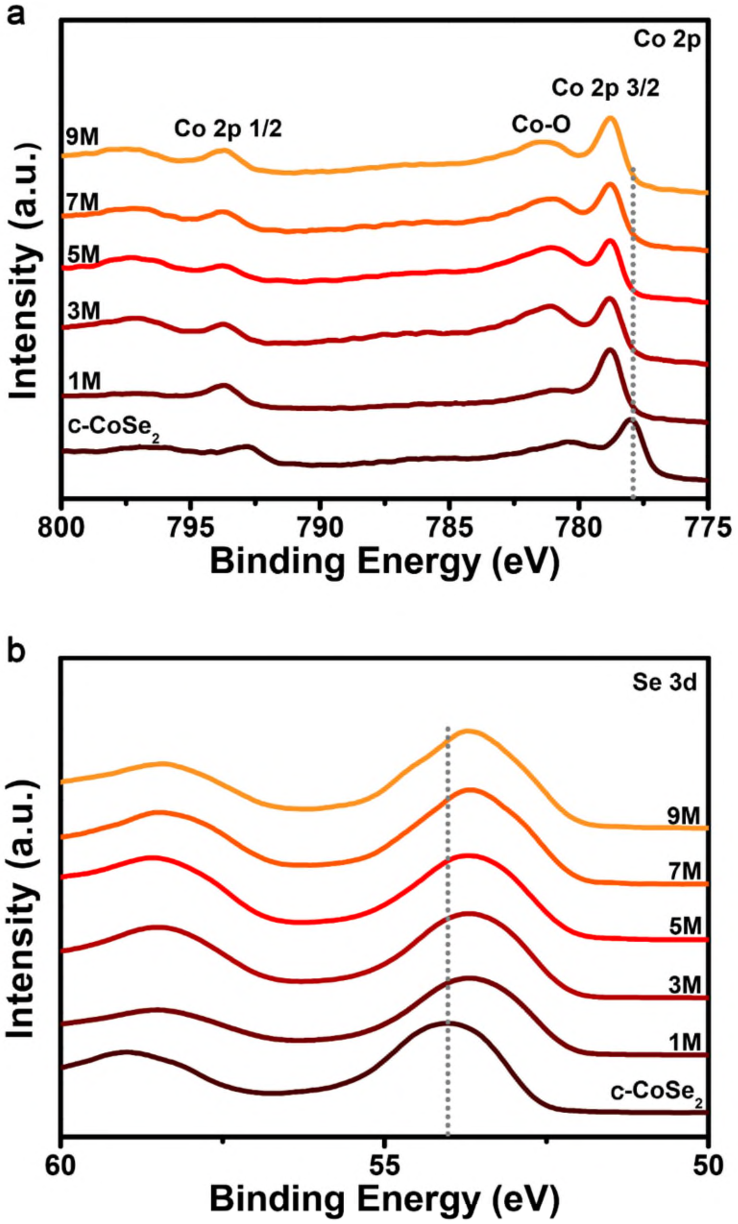

Supplementary Figure 37. XPS spectra. Co *2p* spectra (**a**) and Se *3d* spectra (**b**) of the c- and m-$CoSe_2$ obtained after the syntheses were performed at various KOH concentrations.

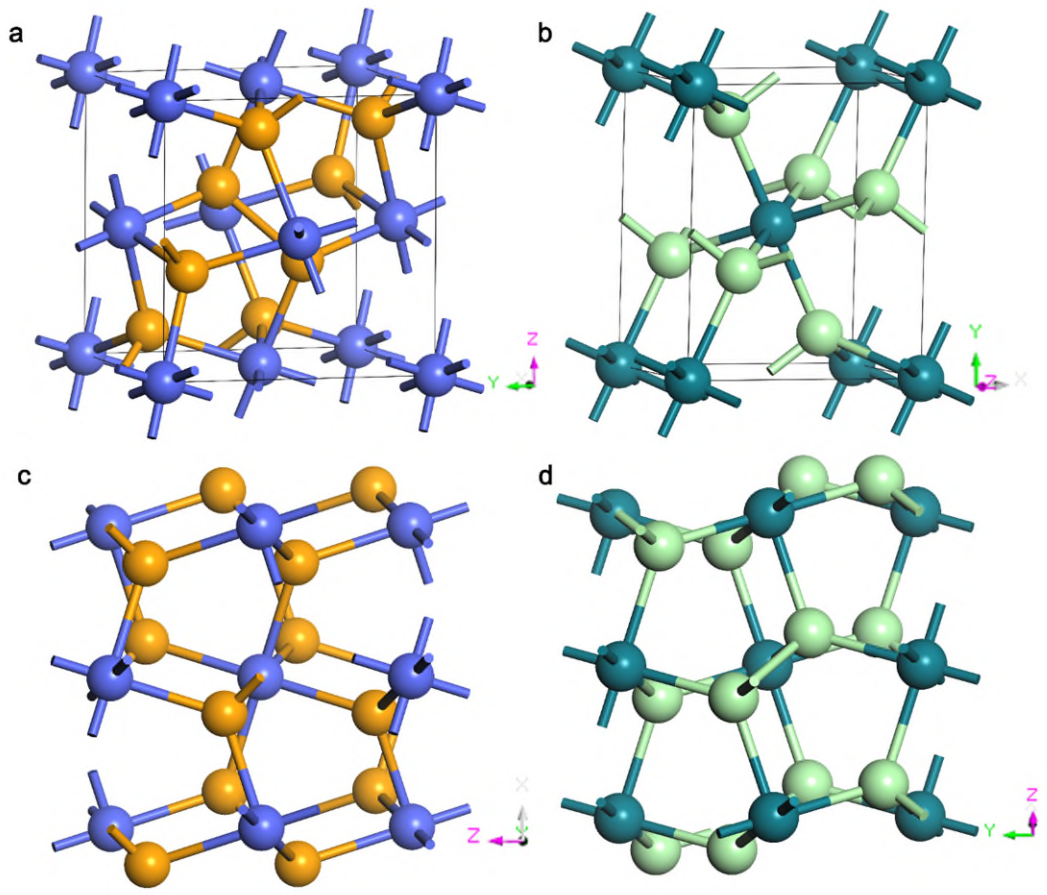

Supplementary Figure 38. Crystal structure. Unit cell of the **a,** c-$CoSe_2$ and **b,** o-$CoSe_2$. Side view of the nonpolar **c,** pyrite (100) surface and **d,** marcasite (101) surface. The atomic layers have the repetitive pattern [Se-Co-Se] along the surface direction. [Se-Se-Co] and [Co-Se-Se] are the other two possible repeating units. In both cases, a polar surface is created. Therefore, the surface composed of [Se-Co-Se] is the only possible nonpolar surface.

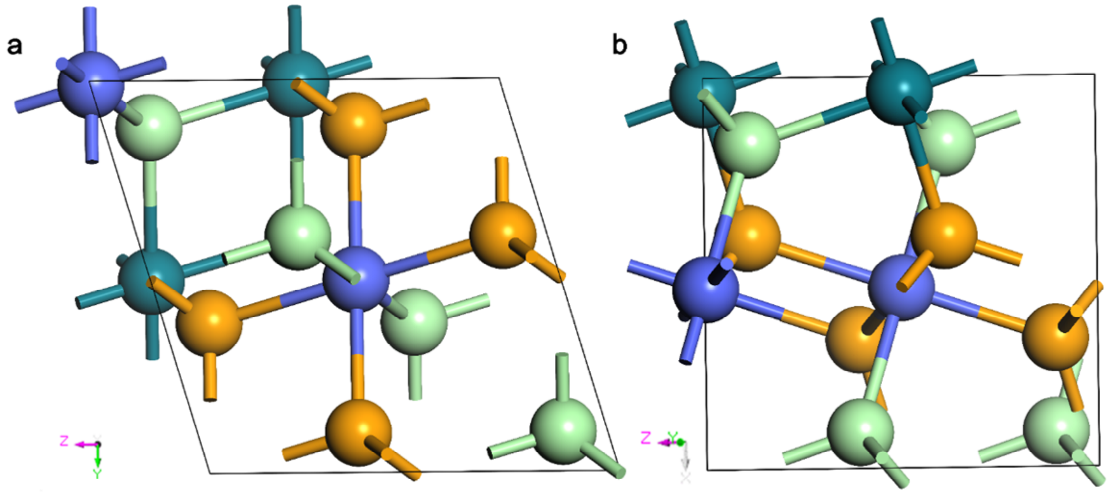

Supplementary Figure 39. Crystal structure of the m-$CoSe_2$. a, The side view and **b,** the top view of the unit cell of the m-$CoSe_2$. The crystal structure of m-$CoSe_2$ is different from that of o-$CoSe_2$ and c-$CoSe_2$, which is a new phase.

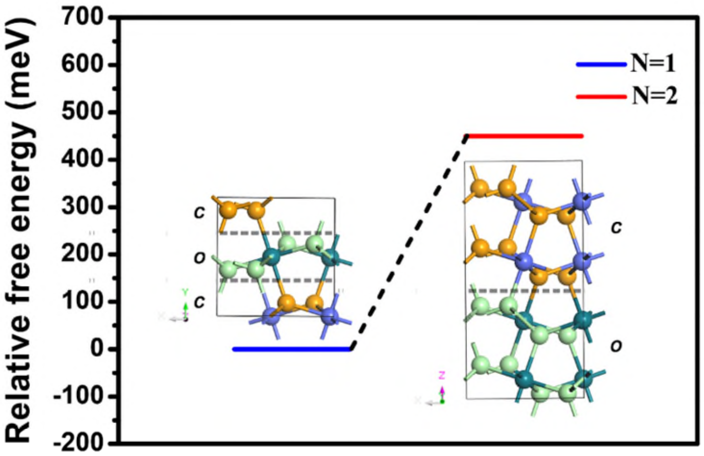

Supplementary Figure 40. The relative free energy of the m-CoSe$_2$. The m-CoSe$_2$ with the difference constituent unit sizes. The blue (red) line represents the unit with one (two) Se-Co-Se patterns. N represents the number of the Se-Co-Se patterns of each phase in the mixed phase. The N = 1 phase is more stable than the N = 2, which means that the size of each phase in the mixed phase is important for the stability of the mixed phase.

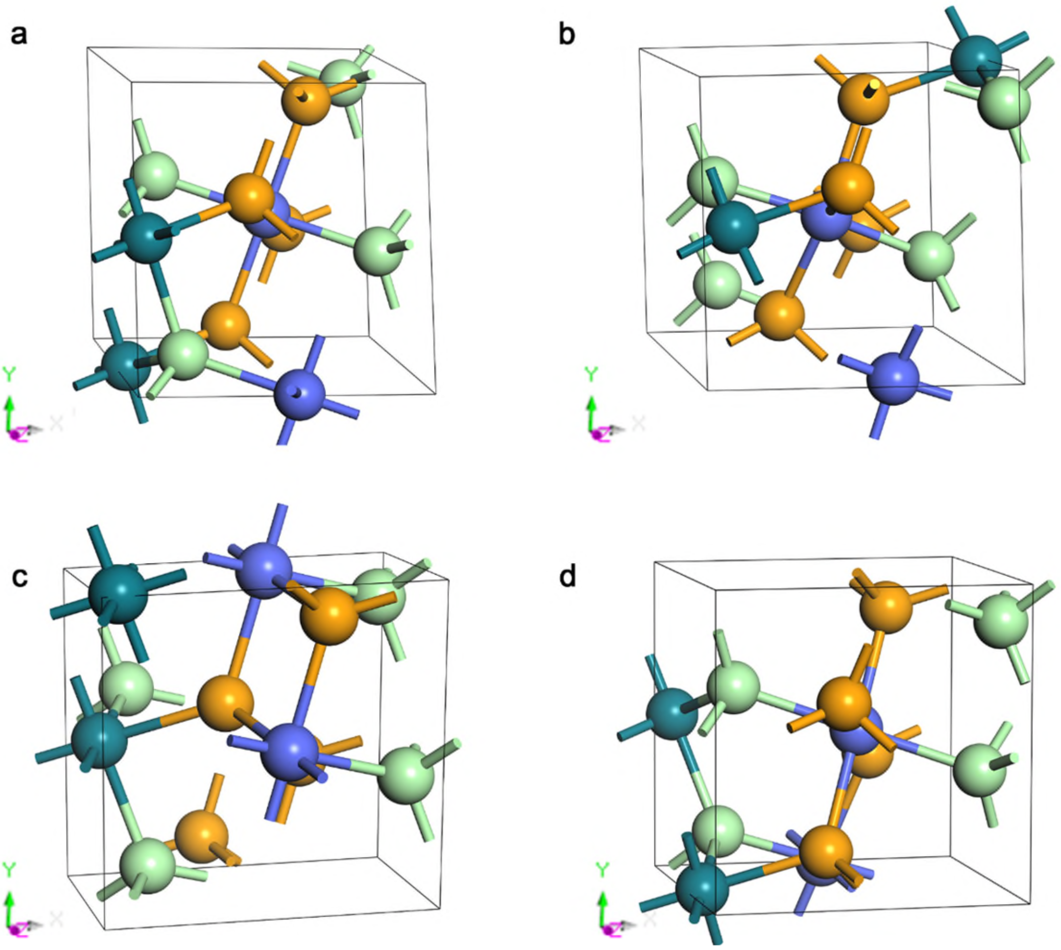

Supplementary Figure 41. Crystal structure of the m-$CoSe_2$. a, N=1, θ=0°. **b,** N=1, θ=90°. **c,** N=1, θ=180°. **d,** N=1, θ=270°. The number of Se-Co-Se layers along the normal direction of the interface shall be denoted as N. We denote the rotation angle as θ. The combination of pyrite (100) surface and martensitic (101) surface can produce a variety of different interfaces, so we create different pyrite (100)-martensitic (101) interfaces depending on the orientation angle θ. We match the two phases, and relative to the pyrite phase, perform an integer multiple of the 90° rotation of the marcasite phase relative to the interface normal direction, resulting in these four structures.

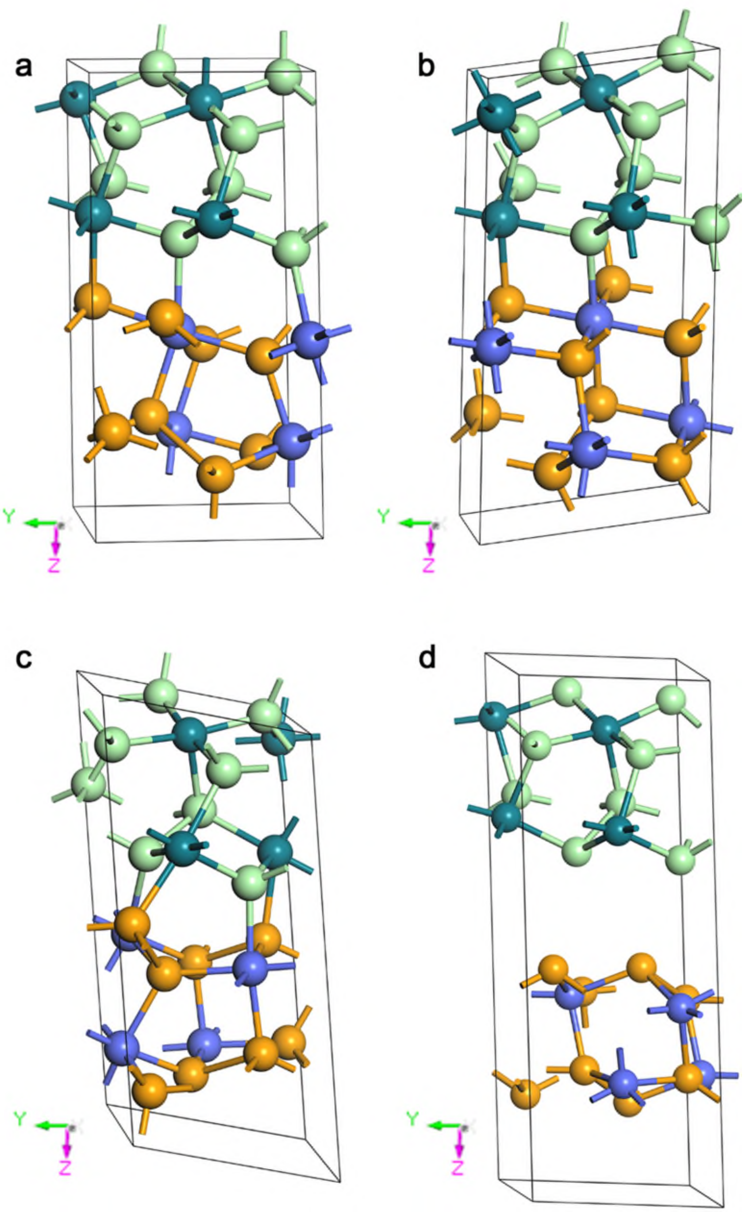

Supplementary Figure 42. Crystal structure of the m-$CoSe_2$. a, N=2, θ=0°. **b,** N=2, θ=90°. **c,** N=2, θ=180°. **d,** N=2, θ=270°. N=2 represents there are two Se-Co-Se patterns of each phase in the mixed phase, θ denotes the rotation angle. We match the two phases, and relative to the pyrite phase, perform an integer multiple of the 90° rotation of the marcasite phase relative to the interface normal direction, resulting in these four structures.

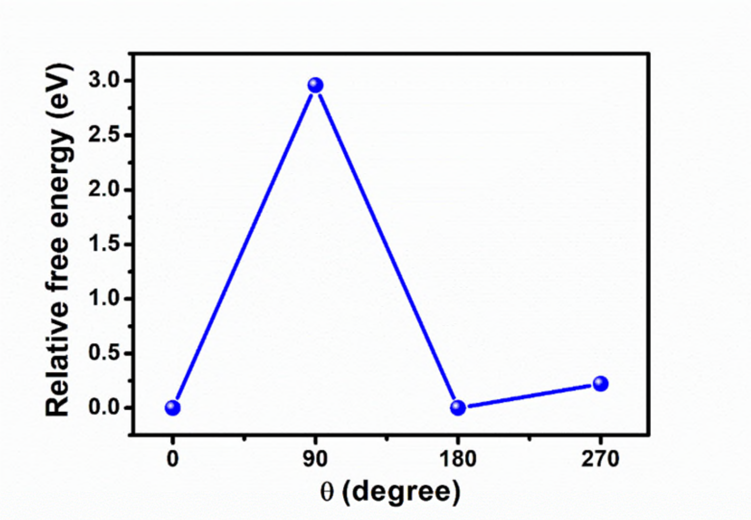

Supplementary Figure 43. The relative free energy of the m-$CoSe_2$ for the N=1. It shows that the free energy of the *m*-$CoSe_2$ depends on the relative orientation between the two phases when N =1. The m-CoSe2 is the most stable when θ=0° or θ=180

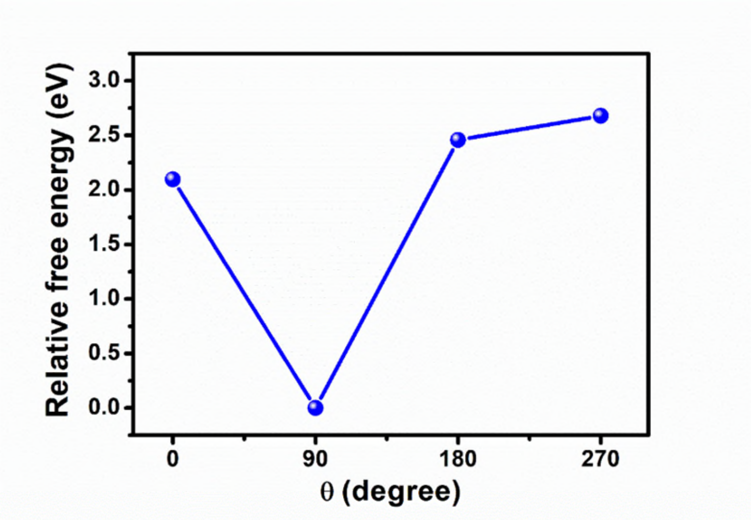

Supplementary Figure 44. The relative free energy of the m-$CoSe_2$ for the N=2. It shows that the free energy of the m-$CoSe_2$ depends on the relative orientation between the two phases when N =2 and its regularity is different from the free energy of the m-$CoSe_2$ when N=1. For the case of N=2, m-$CoSe_2$ is the most stable when θ=90°.

Supplementary References

1 Liu, Q. *et al.* $CoSe_2$ nanowires array as a 3D electrode for highly efficient electrochemical hydrogen evolution. *ACS Appl. Mater. Inter.* **7**, 3877-3881 (2015).

2 Zhang, H. *et al.* Polymorphic $CoSe_2$ with mixed orthorhombic and cubic phases for highly efficient hydrogen evolution reaction. *ACS Appl. Mater. Inter.* **7**, 1772-1779 (2015).

3 Kong, D. *et al.* First-row transition metal dichalcogenide catalysts for hydrogen evolution reaction. *Energy Environ. Sci.* **6**, 3553-3558 (2013).

4 Kong, D., Wang, H., Lu, Z. & Cui, Y. J. J. o. t. A. C. S. $CoSe_2$ nanoparticles grown on carbon fiber paper: an efficient and stable electrocatalyst for hydrogen evolution reaction. *J. Am. Chem. Soc.* **136**, 4897-4900 (2014).

5 McCarthy, C. L., Downes, C. A., Schueller, E. C., Abuyen, K. & Brutchey, R. L. Method for the Solution Deposition of Phase-Pure CoSe2 as an Efficient Hydrogen Evolution Reaction Electrocatalyst. *ACS Energy Lett.* **1**, 607-611 (2016).

6 Kim, J. K., Park, G. D., Kim, J. H., Park, S. K. & Kang, Y. C. Rational Design and Synthesis of Extremely Efficient Macroporous $CoSe_2$ -CNT Composite Microspheres for Hydrogen Evolution Reaction. *Small* **13** (2017).

7 Xiao, H. *et al.* Lamellar structured $CoSe_2$ nanosheets directly arrayed on Ti plate as an efficient electrochemical catalyst for hydrogen evolution. *Electrochim. Acta* **217**, 156-162 (2016).

8 Yue, H. *et al.* Interwoven $CoSe_2$/CNTs hybrid as a highly efficient and stable electrocatalyst for hydrogen evolution reaction. *Electrochim. Acta* **253**, 200-207 (2017).

9 Zhou, W. *et al.* $CoSe_2$ nanoparticles embedded defective carbon nanotubes derived from MOFs as efficient electrocatalyst for hydrogen evolution reaction. *Nano Energy* **28**, 143-150 (2016).

10 Wang, K. *et al.* $CoSe_2$ necklace-like nanowires supported by carbon fiber paper: a 3D integrated electrode for the hydrogen evolution reaction. *J. Mater. Chem. A* **3**, 9415-9420 (2015).
